# Effectiveness of Antimicrobial Photodynamic Therapy in the Treatment of Periodontitis: A Systematic Review and Meta-Analysis of In Vivo Human Randomized Controlled Clinical Trials

**DOI:** 10.3390/pharmaceutics13060836

**Published:** 2021-06-04

**Authors:** Snehal Dalvi, Stefano Benedicenti, Tudor Sălăgean, Ioana Roxana Bordea, Reem Hanna

**Affiliations:** 1 Department of Surgical Sciences and Integrated Diagnostics, Laser Therapy Centre, University of Genoa, Viale Benedetto XV, 6, 16132 Genoa, Italy; stefano.benedicenti@unige.it (S.B.); reemhanna@hotmail.com (R.H.); 2 Department of Periodontology, Swargiya Dadasaheb Kalmegh Smruti Dental College and Hospital, Nagpur 441110, India; 3 Department of Land Measurements and Exact Sciences, University of Agricultural Sciences and Veterinary Medicine Cluj-Napoca, 400372 Cluj-Napoca, Romania; 4 Department of Oral Rehabilitation, “Iuliu Hațieganu” University of Medicine and Pharmacy Cluj-Napoca, 400012 Cluj-Napoca, Romania; roxana.bordea@ymail.com; 5 Department of Oral Surgery, Dental Institute, King’s College Hospital NHS Foundation Trust, London SE5 9RS, UK

**Keywords:** antimicrobial photodynamic therapy, periodontitis, scaling and root planing, systematic review, meta-analysis

## Abstract

This systematic review and meta-analysis evaluated antimicrobial photodynamic therapy (aPDT) efficacy in periodontitis. The review protocol was conducted in accordance with PRISMA statements, Cochrane Collaboration recommendations and is registered in PROSPERO (CRD 42020161516). Electronic and hand search strategies were undertaken to gather data on in vivo human RCTs followed by qualitative analysis. Differences in probing pocket depth (PPD) and clinical attachment level (CAL) were calculated with 95% confidence intervals and pooled in random effects model at three and six months. Heterogeneity was analyzed, using Q and I^2^ tests. Publication bias was assessed by visual examination of the funnel plot symmetry. Sixty percent of 31 eligible studies showed a high risk of bias. Meta-analysis on 18 studies showed no additional benefit in split mouth studies in terms of PPD reduction (SMD 0.166; 95% CI −0.278 to 0.611; *P* = 0.463) and CAL gain (SMD 0.092; 95% CI −0.013 to 0.198; *P* = 0.088). Similar findings noted for parallel group studies; PPD reduction (SMD 0.076; 95% CI −0.420 to 0.573; *P* = 0.763) and CAL gain (SMD 0.056; 95% CI −0.408 to 0.552; *P* = 0.745). Sensitivity analysis minimized heterogeneity for both outcome variables; however, intergroup differences were not statistically significant. Future research should aim for well-designed RCTs in order to determine the effectiveness of aPDT.

## Highlights

1. Limitations of scaling and root planing (SRP) have directed the research to assess alternative comprehensive treatment strategies.
2. Antimicrobial Photodynamic therapy (aPDT) involves photo-excitation of photosensitizer dye upon illumination by a light of a matched wavelength.
3. This systematic review and meta-analysis evaluated the effectiveness of aPDT in the treatment of periodontitis.
4. In spite of the inconsistencies in their findings and methodological bias, the majority of the studies have demonstrated aPDT effectiveness.
5. The efficacy of aPDT in improving treatment outcomes when it is utilized in the non-surgical management of periodontitis remains debatable.

## 1. Introduction

Antimicrobial Photodynamic therapy (aPDT) involves photo-excitation, which occurs when a photosensitizer (PS) dye is illuminated by a light of a matched wavelength, resulting in its activation and stimulation of a phototoxic response in the presence of ambient oxygen [1]. It has been persistently observed that bacterial recolonizations of *Aggregatibacter actinomycetemcomitans (A.a)* occur in periodontal pockets even after scaling and root planing (SRP) [2]. Aggressive periodontitis (AgP) is frequently associated with fewer local etiologic factors; therefore, it is believed that the affected patients are more likely to benefit from the antimicrobial effect of aPDT [3]. In contrast, chronic periodontitis (CP) patients usually have complex and thick deposits of polymicrobial communities on the affected root surfaces [4]. This may hamper penetration of PS, thereby reducing its effect and leading to an increase in the ‘red complex’ bacterial counts within a short period of time, resulting in a disease relapse [5]. Hence, the concept of replacing conventional SRP with aPDT is a controversial one with several imperative demerits, as enlisted above.

Utilization of adjunctive aPDT and its comparison with the gold standard SRP is a concept that has been studied extensively in both CP and AgP patients [6,7,8,9,10,11,12,13,14,15,16]. While SRP can quantitively lower the biomass of bacteria, aPDT has a more qualitative approach of a non-invasive nature, by creating alterations in cell membranes or Deoxyribonucleic Acid (DNA) damage [5]. Hence, a combination of these two therapies can be vouched for, since their mechanisms of action on microbiota and role in the periodontal repair process is distinct from the other and thus might have synergistic effects [17]. 

Distant sites of infection such as tonsils or base of tongue, which are affected due to the spread of tissue penetrating periopathogens, can be successfully reduced with local or systemic antibiotics (AB) [18]. Nonetheless, many clinicians often conduct NSPT without adjunctive AB, which is only used when initial treatment has failed [19]. In AgP, evidence suggests that SRP+AB therapy does not show satisfactory long-term results, unless re-instrumentation of affected sites is performed, as an additional step in the maintenance phase [20]. Furthermore, owing to the development of antibiotic resistant strains, it has been suggested that AB usage should be restricted to those with a highly active disease or a specific microbiological profile [21]. In order to maintain an adequate mean inhibitory concentration (MIC) of any antimicrobial drug, either a sustained-release carrier medium is required or, conversely, a prompt bactericidal approach is needed to overcome the problem of physical displacement from the sulcus [22]. The aPDT falls into the latter category, demonstrating a 4--6-fold logarithmic bacterial reduction within a time frame of 60 s along with repeated applications [23]. A comprehensive assessment to evaluate the impact of these new trials on the role of aPDT in the treatment of periodontitis is unresolved, owing to the diversity in the methodology and results of existing scientific evidence [6,7,8,9,10,11,12,13,14,15,16].

In lieu of the prevailing pertinent literature, the present systematic review and meta-analysis aimed to provide a systematic evaluation of available scientific evidence to determine the efficacy of aPDT in the treatment of periodontitis. The objectives of this critical review were to evaluate the outcomes of this treatment strategy through various PS-laser wavelength combinations, as well as the laser parameters, in order to deduce an ideal PS-laser wavelength combination and treatment protocol for future scientific research. 

## 2. Materials and Methods

### 2.1. Protocol and Registration

The present systematic review was reported based on the guidelines of Preferred Reporting Items for Systematic Reviews and Meta-Analysis (PRISMA) Statement and Cochrane Collaboration recommendations (Supplementary file 1) [24,25]. The review protocol is published in the Prospective Register of Systematic Reviews (PROSPERO); ref CRD 42020161516. 

### 2.2. Population (P), Intervention (I), Comparison (C) and Outcomes (O)—PICO

- **Population:** Patients diagnosed with Periodontitis (CP or AgP) [26]
- **Intervention:** Utilisation of aPDT as a monotherapy or as an adjunct to SRP
- **Comparison:** Utilisation of SRP alone or SRP with adjunctive AB therapy
- **Outcome:** Evaluation of clinical and/or microbiological and/or immunological profiles

### 2.3. Focused Research Question

Is aPDT effective as a primary mode of treatment or as an adjunct to SRP compared to SRP alone or in combination with local or systemic antibiotics (AB), in terms of clinical or microbiological or immunological profiles, in patients with Periodontitis?

### 2.4. Search Strategy

The search strategy only included terms relating to or describing the study’s domain and intervention. The use of relevant free text keywords and medical subject heading (Mesh) terms, which were logically connected with the help of Cochrane MEDLINE filters for controlled trials of interventions, was implemented. Individual search algorithms were developed for the following databases: MEDLINE (NCBI PubMed and PMC), Cochrane Central Register of Controlled Trials (CCRCT), Scopus, ScienceDirect, Google Scholar, EMBASE and EBSCO. Electronic search databases were searched thoroughly from their earliest records until 31 December 2019. The following journals were manually searched: Journal of Periodontology, Photomedicine and Laser Surgery, Clinical Oral Investigation, Journal of Clinical Periodontology, Journal of Dental Research, Lasers in Medical Science, Journal of Photochemistry and Photobiology and Photodiagnosis and Photodynamic Therapy. Related review articles and reference lists of all identified articles were searched through for further studies. Abstracts of the American Academy of Periodontology (AAP) and the European Federation of Periodontology (EFP) as well as sources for grey literature were screened to detect unpublished studies. In some instances, an attempt was made to establish a communication with the corresponding author in an attempt to obtain additional information related to the study; however, the attempts were unsuccessful. Search strategy was performed by two blinded, independent reviewers (S.D. and R.H.). In order to assess inter-reviewer reliability analysis, Kappa (κ) statistics were performed and a minimum value of 0.8 was considered acceptable [27]. In case of any disagreements, reviewers would discuss the discrepancies with a third author (S.B.), if necessary. 

### 2.5. Search Algorithms

“Photodynamic therapy” **OR** “photochemotherapy” 

**AND**

“Scaling” **OR** “Root planing” **OR** “non-surgical periodontal therapy” 

**AND**

“Periodontitis” **OR** “Chronic Periodontitis” **OR** “Aggressive Periodontitis” **OR** “Early Onset Periodontitis” 

### 2.6. Eligibility Criteria 

#### 2.6.1. Inclusion Criteria

1. Subjects diagnosed with CP or AgP according to 1999 AAP Classification of Periodontal diseases and conditions [26].
2. Studies included: In vivo human RCT’s comparing the efficacy of aPDT in CP or AgP as monotherapy or adjunctive to SRP compared to SRP alone or in combination with AB.
3. Parallel group (PG) and split-mouth (SM) studies.
4. Age group >18 years, fit and healthy subjects.
5. No language restrictions for search strategy.
6. Studies that have utilized any PS dye (regardless dose and incubation period) and laser wavelength combination.
7. Studies reporting at least one of the following parameters as an outcome variable: probing pocket depth (PPD), loss of clinical attachment level (CAL), bleeding on probing (BOP), plaque index (PI), gingival index (GI), microbiological profile, or immunological profile.
8. Studies with a minimum follow-up period of at least one month after treatment.

#### 2.6.2. Exclusion Criteria

1. Subjects with systemic diseases or on medications that can influence the outcome variables.
2. Subjects who have undergone any periodontal therapy and/or antibiotic therapy in the last six months prior to RCT enrolment.
3. Studies utilizing low level laser therapy or laser therapy alone, as one of the intervention groups as compared to aPDT.
4. Studies involving utilization of aPDT for residual pockets or in supportive periodontal therapy (SPT).
5. Studies that have utilized light emitting diodes (LEDs) as a light source.
6. No outcome variable of interest.
7. Pregnancy.
8. Smoking.
9. Narrative and systematic reviews, in vitro studies, in vivo animal studies, commentaries, interviews, updates, case series and case reports.

### 2.7. Systematic Review Outcomes

#### 2.7.1. Primary Outcome Measures

Changes in PPD and CAL from baseline up to the end of follow-up.

#### 2.7.2. Secondary Outcome Measures 

Changes in GR, BOP, PI, GI, microbiological and immunological profile from baseline up to the end of follow-up.

### 2.8. Data Extraction

Two reviewers independently (S.D. and R.H.) selected eligible studies from the search. They performed the review, assessment and data extraction for each eligible study. Each study received an identification with the name of the first author, year of publication and origin. A tabular representation of additional relevant information such as impact factor of journal, study design, sample size, demographics of the participants, baseline characteristics, intervention and comparator groups, type of photosensitizer used and dosage, laser parameters utilized, number of aPDT sessions performed, follow-up duration, statistical tests performed and results and conclusions, were gathered from each eligible study.

### 2.9. Qualitative Analysis

A qualitative assessment for each study was performed using the Revised Cochrane Risk-of-Bias (RoB) tool for Randomized trials, Version 2.0 (RoB 2) by two independent reviewers (S.D. and R.H.) [28,29,30]. Detailed assessment under the following headings was performed: 1. Bias arising from the randomization process; 2. Bias due to deviations from intended interventions; 3. Bias due to missing outcome data; 4. Bias in measurement of the outcome; 5. Bias in selection of the reported result. Depending upon fulfilment of above-mentioned criteria, the studies were determined as low, moderate or high RoB. Disagreements between the reviewers were resolved by discussion with a third author (S.B.) as well as use of ‘discrepancy check’ feature in RoB 2, in order to obtain consensus. 

### 2.10. Statistical Analysis of Data

When appropriate and quantifiable data of interest were extracted from the eligible studies and combined for meta-analyses, using Stata version 15.1 software (StataCorp, Pyrmont, Australia), random effects meta-analyses were conducted to reflect the expected heterogeneity. As continuous outcomes were expected, overall treatment effects were calculated through pooled standardized mean differences (SMDs) with associated 95% confidence intervals (95% CIs) for PPD and CAL. When information was presented in median and inter-quartile ranges, means and SDs were estimated [31]. Results from SM and PG studies were pooled separately at 3 and 6 months, respectively. A pooled overall effect was considered statistically significant when *p* < 0.05. Consequently, statistical heterogeneity to identify outlier studies was performed by visual inspection of forest plots. Additionally, the Cochran Q test was conducted to assess statistical heterogeneity (*p* < 0.10) [32]. *I*^2^ statistics for homogeneity was expressed in a range of 0–100%, with the following interpretation; 0% = no evidence of heterogeneity; 30–60% = moderate heterogeneity; 75–100% = high heterogeneity [33]. Sensitivity analysis was conducted to negate the effect of heterogeneity in between included studies by identifying the outlier studies by visual inspection of forest plots [34]. Publication bias was evaluated by visual assessment of funnel plot symmetry.

## 3. Results

### 3.1. Study Selection

**Figure 1:**
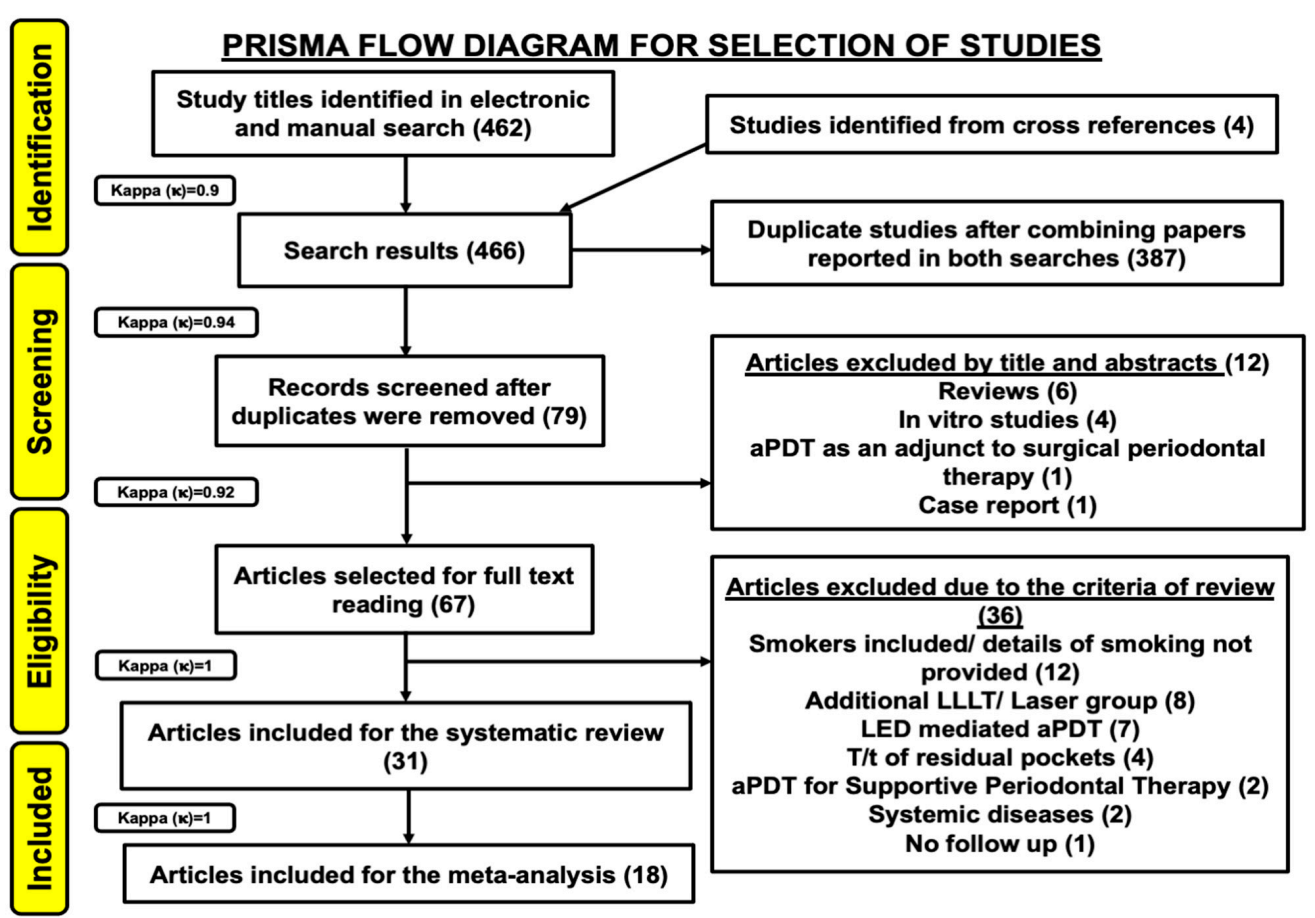
PRISMA flow diagram of the study selection criteria.

Four hundred and sixty-two study titles were obtained from a combined electronic and manual search. Four study titles were obtained from cross-references. Therefore, a total of 466 study titles were included from all databases in the preliminary screening (inter-reviewer agreement, κ = 0.9). Three hundred and eighty-seven articles were excluded, due to duplication and the remaining 79 records were further evaluated (inter-reviewer agreement, κ = 0.94). Twelve articles were excluded based on their titles and abstracts, mainly due to an inappropriate study design (inter-reviewer agreement, κ = 0.92). Thus, 67 articles were assessed for their eligibility. These articles were evaluated based on eligibility criteria. Additionally, 36 studies were excluded due to following reasons: Smokers were included or smoking details were not provided in 12 studies [23,35,36,37,38,39,40,41,42,43,44,45]; Laser or LLLT was utilized, as an adjunct to SRP in eight studies [46,47,48,49,50,51,52,53]; LED-aPDT was performed in seven studies [54,55,56,57,58,59,60]; aPDT was used in management of residual pockets in four studies [61,62,63,64] and as an adjunct to supportive periodontal therapy in two studies [65,66]; patients with systemic diseases were included in two studies [67,68], whereas one study did not perform a follow-up assessment [69] (inter-reviewer agreement, κ = 1). Hence, out of 67 full text articles, 31 articles were included and analyzed in the present systematic review [2,3,5,17,70,71,72,73,74,75,76,77,78,79,80,81,82,83,84,85,86,87,88,89,90,91,92,93,94,95,96]. All included articles were in vivo human studies. A meta-analysis on 18 out of 31 studies which assessed efficacy of SRP+aPDT was conducted [17,71,73,74,75,76,80,81,84,85,86,88,89,90,91,92,93,96] (inter-reviewer agreement, κ = 1). Figure 1 depicts the PRISMA flow diagram for search strategy utilized in the present systematic review and meta-analysis. 

### 3.2. Study Characteristics

#### 3.2.1. Country of Origin

A substantial diversity in the country of origin was noted amongst included papers (Table 1). Distribution of studies was as follows: 11 in Brazil [2,3,5,17,71,82,83,87,92,95,96], 6 in India [78,81,88,89,91,93], 4 in Germany [76,77,80,94], 4 in Iran [70,85,86,90], 3 in Poland [72,73,74], whereas there is 1 study each, in the following countries; Spain [75], Japan [79], Thailand [84].

#### 3.2.2. Study Design 

Twenty studies were conducted using a SM study design [2,3,5,17,70,71,77,80,81,82,84,85,86,87,89,90,92,93,94,95], whereas a PG study design was utilized in the remaining 11 studies [72,73,74,75,76,78,79,83,88,91,96] (Table 1).

#### 3.2.3. Selection Criteria

Several inconsistencies were observed amongst the included studies [2,3,5,17,70,71,72,73,74,75,76,77,78,79,80,81,82,83,84,85,86,87,88,89,90,91,92,93,94,95,96], which have been outlined in Table 1, in which 21 out of 31 studies included patients with CP [17,70,75,76,77,78,79,80,81,82,84,86,88,89,90,91,92,93,94,95,96], whereas the remaining 10 studies included patients with AgP [2,3,5,71,72,73,74,83,85,87]. 

#### 3.2.4. Documentation of Laser Parameters

**Figure 2:**
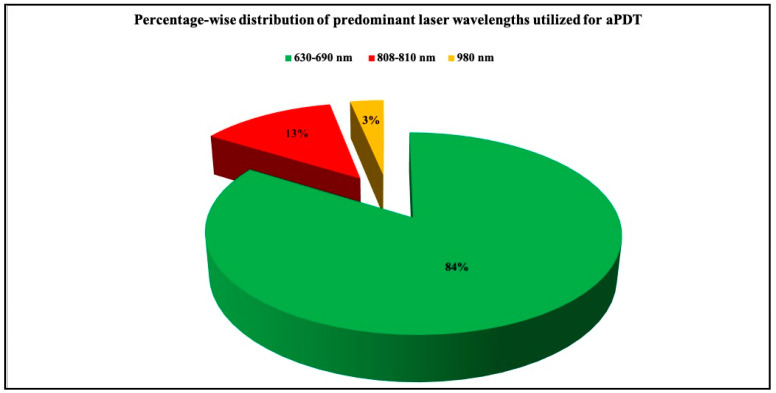
3D pie diagram illustrating the percentage-wise distribution of predominant laser wavelengths utilized for aPDT in the included studies.

Table 2 describes various dye laser combinations, as well as laser dosimetry that was utilized to perform aPDT in all eligible studies. Twenty-six out of 31 studies utilized a laser wavelength in the range of 630–690 nm [2,3,5,17,70,71,72,73,74,75,76,77,79,81,82,83,84,85,86,87,88,91,92,94,95,96] to perform aPDT. While four studies utilized a laser wavelength in the range of 808–810 nm [78,80,90,93], one of the included studies utilized a 980 nm diode laser wavelength to perform aPDT [89] (Table 2) (Figure 2). Emission mode was reported only in five studies [78,88,90,92,93], in which four of them utilized a continuous wave emission mode [78,88,90,92], whilst the remaining one study utilized a gated continuous wave emission mode [93]. Eighteen out of the 31 eligible studies used the laser fibre tip in ‘contact mode’ with the periodontal pocket in order to perform aPDT [2,3,5,78,81,82,84,85,86,88,89,90,91,92,93,94,95,96]. Only 5 studies reported total energy, and it ranged from 1.5–9 J [82,90,92,93,95,96]. Only 19 studies reported power output in the range of 30 mW–1 W [71,75,76,77,78,79,80,82,83,84,85,87,89,90,91,92,93,94,95,96], whereas the use of a power meter to measure the therapeutic power output, reaching the target tissues was not performed in any of the included studies. Spot size was reported in only four studies [5,85,92,96] ranging from 0.02–0.07 cm^2^. Ten out of the 31 studies reported the diameter of fibre tip, [2,3,5,80,81,82,88,89,93,94] ranging from 200–600 μm. The energy density (fluence) was calculated in 18 out of 31 studies [5,17,70,71,72,73,74,78,79,80,82,83,86,87,92,93,95,96], and its value ranged from 0.01–2829 J/cm^2^, whereas the power density (irradiance) values ranged from 60 mW–4 W/cm^2^ and were calculated in 13 studies [2,3,5,17,71,72,73,74,81,88,92,94,95]. Finally, the exposure time for laser irradiation was mentioned in all included studies except one study [80], and the values ranged from 10–120 s/site amongst included studies.

#### 3.2.5. PS Utilized

**Figure 3:**
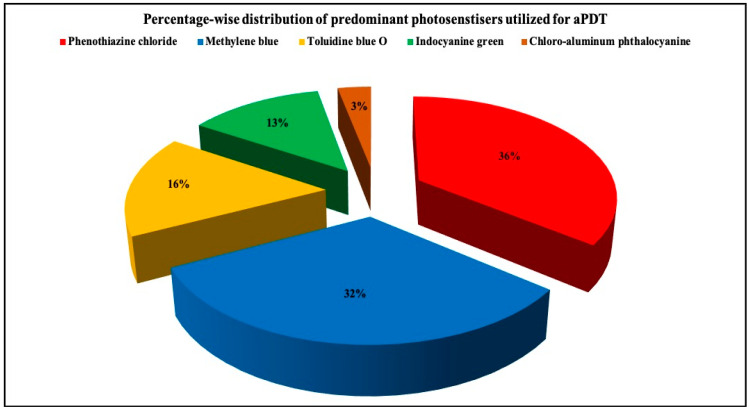
3D pie diagram illustrating the percentage-wise distribution of predominant photosensitizers utilized for aPDT in the included studies.

Type of PS varied amongst eligible clinical trials. Eleven studies utilized phenothiazine chloride [2,3,5,71,72,73,74,76,81,84,94] while 10 employed methylene blue [17,75,77,79,82,87,88,89,96]. Five studies utilized toluidine blue O [70,85,86,91,95], four studies used indocyanine green [78,80,90,93], whereas chloro-aluminum phthalocyanine was utilized in one study [92] (Figure 3). Interestingly, 18 out of 31 studies specified the concentration of the PS [2,3,17,71,75,77,78,79,80,82,83,87,88,89,90,92,95,96], while 13 studies failed to report the same [5,70,72,73,74,76,81,84,85,86,91,93,94] (Table 2).

#### 3.2.6. Utilization of aPDT as a Mono-Therapeutic or an Adjunctive Therapeutic Agent

While 28 out of the 31 eligible studies utilized SRP+aPDT, aPDT monotherapy was performed in three studies [2,3,5] (Table 1). 

#### 3.2.7. Comparison in between SRP+ aPDT versus SRP+AB

Six out of the 31 eligible studies compared efficacy of SRP+aPDT versus SRP+AB [72,73,74,79,83,96] (Table 1).

#### 3.2.8. Number of aPDT Sessions

While a single session of aPDT was applied in 22 out of the 31 included studies [2,3,5,70,76,77,78,80,81,82,83,84,85,86,87,88,89,91,92,93,94,95], multiple aPDT sessions were performed in nine studies [17,71,72,73,74,75,79,90,96]. None of the eligible studies compared single versus multiple sessions of aPDT (Table 2).

#### 3.2.9. Follow-Up Assessment

A follow-up assessment at three months from the baseline visit was performed in 18 out of the 31 eligible studies [2,3,5,17,70,71,74,76,81,85,86,87,90,91,92,93,94,96], whereas 12 studies conducted a longer follow-up assessment at six months [72,73,75,77,78,80,82,83,84,88,89,95]. Only one study performed a follow-up assessment at one month from the baseline visit [79]. A long-term follow-up of a minimum one year from baseline visit lacked in all eligible studies.

### 3.3. Qualitative Assessment

**Figure 4:**
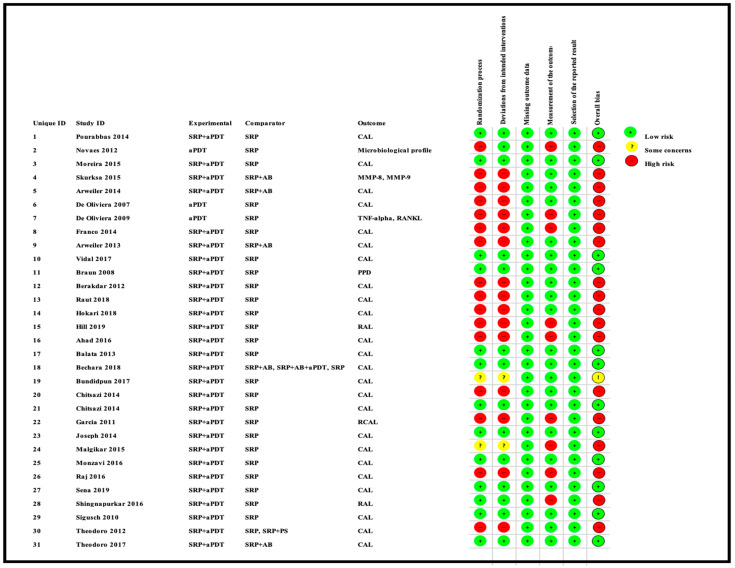
Risk of Bias assessment summary of the included studies based on the consensual answers across two individual assessors (S.D. and R.H.).

**Figure 5:**
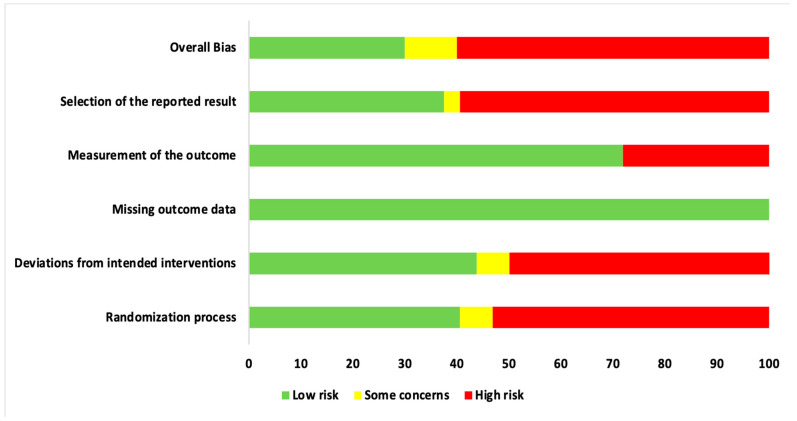
Risk of Bias assessment graph of the included studies expressed as percentages, based on the consensual answers across two individual assessors (S.D. and R.H.).

Qualitative assessment was performed using the RoB 2 tool, designed for in vivo human RCTs, as depicted in Figure 4 and Figure 5. The most recent version of this tool was utilized to perform a qualitative assessment for both randomized PG and SM human RCTs [29,30]. Figure 4 represents a risk of bias assessment summary of all eligible studies. Figure 5 is a graphical representation of percentage RoB score for each risk domain, which has been evaluated, using the abovementioned tool. Furthermore, 53.1% of included trials were at a high risk of inadequate randomization, whereas 40.6% and 6.3% of included trials were at a low risk or had some concerns, respectively. In addition, 50% of included studies were at a high risk of deviations from intended interventions, whereas 43.7% and 6.3% of them were at a low risk or had some concerns, respectively. All included papers reported substantial evidence (100%) for reporting missing outcome data and, hence, were at a low risk. Although a majority of studies were free of bias arising from reporting outcome measurement (71.9%), 28.1% were at a high risk. In terms of selective reporting of the results, inferences are as follows: 59.4% studies were at a high risk, 37.5% studies were at a low risk, and 3.1% studies had some concerns. Overall, 60% studies reported a high risk of bias, while 35% studies had a low risk of bias, and the final 5% studies had some concerns. It should be noted that information provided in these figures represents the consensual answers verified using the ‘Discrepancy check’ feature of RoB 2 tool, across two independent reviewers (S.D. and R.H.) (inter-reviewer agreement, κ = 0.94), and, in case of any disagreements, a third author (S.B.) was consulted.

### 3.4. Quantitative Assessment

#### 3.4.1. Outcome Variables

Primary outcomes of 18 out of 31 studies, which have assessed efficacy of SRP+aPDT in the management of periodontitis, contributed to this meta-analysis [17,71,73,74,75,76,80,81,84,85,86,88,89,90,91,92,93,96]. Data were pooled separately for SM and PG studies for differences in PPD and CAL respectively at three and six months, respectively. At three months, the mean difference in PPD reduction was not statistically significant for SM studies (SMD 0.166; 95% CI −0.278 to 0.611; *P* = 0.463) and PG studies (SMD 0.076; 95% CI −0.420 to 0.573; *P* = 0.763) along with a high heterogeneity for SM studies (Q = 15.81; *P* = 0.0001; I^2^ = 91.21%) and moderate heterogeneity (Q = 11.87; *P* = 0.018; I^2^ = 66.31%) for PG studies (Table 3). The mean difference in PPD reduction at six months did not show a statistically significant difference for SM studies (SMD 0.005; 95% CI –0.126 to 0.136; *P* = 0.935) as well as PG studies (SMD 0.141; 95% CI −1.007 to 1.288; *P* = 0.809) although contrasting findings were noted in terms of level of heterogeneity which was not evident for SM studies (Q = 0.06; *P* = 0.99; I^2^ = 0.00%) and high for PG studies (Q = 18.71; P = 0.0001; I^2^ = 89.31%) (Table 4). CAL gain at three months was not statistically significant in SM studies (SMD 0.092; 95% CI −0.013 to 0.198; *P* = 0.088) with no evident heterogeneity (Q = 8.74; *P* = 0.655; I^2^ = 0.00%) as well as in PG studies (SMD 0.056; 95% CI −0.408 to 0.552; *P* = 0.745) with moderate heterogeneity (Q = 8.95; *P* = 0.028; I^2^ = 70.31%) (Table 3). At six months, results for SM studies were not statistically significant (SMD −0.013; 95% CI −0.148 to 0.121; *P* = 0.846) with no evident heterogeneity (Q = 0.03; *P* = 0.984; I^2^ = 0.00%), whereas, for PG studies, the findings were statistically significant (SMD -0.441; 95% CI −0.805 to −0.075; *P* = 0.018) with no evidence of heterogeneity (Q = 1.70; *P* = 0.42; I^2^ = 0.00%) but favoring control group (Table 4).

Assessment of secondary outcome variables was conducted in the majority of included studies, which are as follows: Changes in GR, BOP, PI and GI in 28 studies [3,17,70,71,73,74,75,76,77,78,79,80,81,82,83,84,85,86,87,88,89,90,91,92,93,94,95,96], microbiological analysis in 11 studies [5,71,75,78,79,80,85,86,91,94,95], and immuno-histological in seven studies [2,17,70,71,72,75,79]. Table 5 provides an overview of clinical parameters which have been assessed in 28 of 31 included studies along with corresponding level of significance, in accordance to data provided in Table 1. Eleven studies performed a microbiological analysis [5,71,75,78,79,80,85,86,91,94,95], out of which five studies reported that aPDT therapy could significantly reduce periopathogenic burden [71,75,78,91,94] and six studies failed to achieve this outcome [5,79,80,85,86,95]. In terms of immune-histological analysis, seven studies [2,17,70,71,72,75,79] assessed various pro-inflammatory cytokines and growth factors such as; IL-1β, IL-10, IF-γ, TNF-α, MMP-8, MMP-9, RANK, RANK-L, OPG and FGF-2 (Table 1). Biomarkers for assessment of bone resorption (RANK, RANK-L, OPG) were assessed in three studies [2,17,75]. Two studies [17,75] assessed efficacy of SRP+aPDT in comparison to conventional SRP alone, and showed that SRP+aPDT successfully suppressed the bone resorption process. Levels of IL-1β, IL-10, IF-γ, and TNF-α were assessed in three studies [70,71,79], of which two studies have confirmed immunological benefits of aPDT [70,71], whereas one study [79] failed to show any advantage of aPDT for the same. It should, however, be noted that, while the former two studies [70,71] have compared the efficacy of SRP+aPDT to conventional SRP alone, the latter study [79] has compared SRP+aPDT to SRP+AB and demonstrated the advantages of AB over aPDT. Additionally, SRP+aPDT showed an increased expression of FGF-2, which plays a role in tissue repair as compared to SRP alone, and was assessed in only one study [17]. A meta-analysis on secondary outcomes was not possible due to disparity in scoring methodology, incomplete, or incomparable data.

#### 3.4.2. Sensitivity Analysis 

A sensitivity analysis was conducted due to the noteworthy heterogeneity arising from outlier studies which were detected upon visual inspection of Forest plots [74,86,90,92,93] (Table 3 and Table 4). This analysis was conducted only for the three-month follow-up due to unavailability of data in included studies (Table 6). In terms of PPD reduction, SM studies (SMD 0.282; 95% CI −0.286 to 0.624; *P* = 0.153) as well as PG studies (SMD 0.257; 95% CI −0.230 to 0.683; *P* = 0.361) did not report a statistically significant improvement. No evident heterogeneity (Q = 9.14; *P* = 0.7; I^2^ = 0.00%) in SM studies and in PG studies (Q = 8.87; *P* = 0.22; I^2^ = 0.00%) was noted (Table 6). Although improvement in CAL gain was noted after omitting outlier studies, this difference was statistically not significant in both SM (SMD 0.162; 95% CI −0.326 to 0.406; *P* = 0.166) and PG studies (SMD 0.227; 95% CI −0.420 to 0.673; *P* = 0.234) with no evident heterogeneity (Q = 8.40; *P* = 0.625; I^2^ = 0.00%) in SM studies as well as in PG studies (Q = 9.7; *P* = 0.22; I^2^ = 0.00%) (Table 6). 

#### 3.4.3. Publication Bias

**Figure 6:**
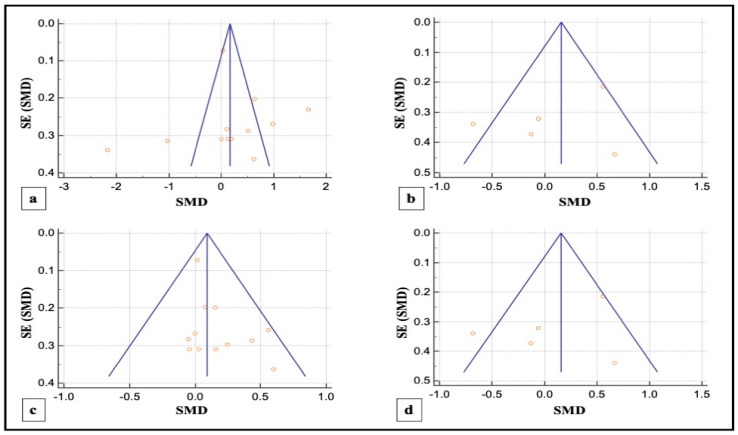
(**a**,**b**) Funnel plots illustrating the publication bias in overall PPD reduction in SM and PG studies, respectively; (**c**,**d**) funnel plots illustrating the publication bias in overall CAL gain in SM and PG studies, respectively. Each circle represents a single included study, the y-axis and x-axis represent the standard error of the effect estimate and the results of the study respectively and the graphical plot resembles an inverted funnel with scatter due to sampling variations.

Visual inspection of funnel plots revealed noticeable asymmetry in SM-study analysis for PPD reduction indicating a probable risk of publication bias in SM studies included in this meta-analysis. However, slight asymmetries for PG studies were noted in corresponding funnel plots suggestive of a low risk of publication bias in the same (Figure 6).

## 4. Discussion

Based on the hypothesis that aPDT monotherapy or as an adjunct to NSPT can enhance the clinical or microbiological or immunological profile in comparison to conventional SRP, or SRP+AB, a critical appraisal of the available scientific evidence was conducted. After meticulous scrutiny, 31 studies were included in the present systematic review [2,3,5,17,70,71,72,73,74,75,76,77,78,79,80,81,82,83,84,85,86,87,88,89,90,91,92,93,94,95,96]. Owing to the methodological discrepancies, only 18 out 31 studies were eligible for a meta-analysis [17,71,73,74,75,76,80,81,84,85,86,88,89,90,91,92,93,96]. This report is the first to evaluate the role of SRP+aPDT compared to SRP alone or SRP+AB in SM and PG studies in AgP as well as in CP patients. The results of this meta-analysis indicated that, in comparison to SRP alone or SRP+AB, SRP+aPDT failed to show any additional benefit in the management of periodontitis up to six months. A significant heterogeneity was reported, arising from confounders in aPDT protocols. Subsequently, after omitting outlier studies [74,86,90,92,93], sensitivity analysis was able to eliminate heterogeneity completely but failed to report statistically significant improvements in primary outcome variables. Furthermore, risk of publication bias was reported indicating a possible selective outcome reporting in eligible published studies. In some instances, missing outcomes could not be detected by comparing the published report with the respective study protocol due to unavailability of the latter. Until now, seven meta-analyses have been reported to assess the role of aPDT in periodontitis [6,7,8,9,10,11,12]. The present review protocol is in accordance with the existing reviews [8,10,11,12]. Azaripour et al. is the only other systematic review and meta-analysis that has assessed the efficacy of SRP+aPDT as compared to SRP alone for SM and PG studies [8]. While three reviews report short-term benefits of aPDT up to 6 months [7,8,9], four have reported otherwise [6,10,11,12]. Our findings are in accordance with findings of the latter scientific reports. In order to gain an insight on merits and inadequacies of each included study, a comprehensive and systematic investigation was performed as follows:

### 4.1. Role of Baseline Characteristics

A key feature of RCTs is the application of balanced baseline characteristics in treatment arms of the trial in order to achieve unbiased treatment outcomes [97]. Most often, researchers provide a tabular representation of relevant variables to confirm an impartial baseline evaluation. In case of missing information on baseline characteristics, a ‘selection bias’ can be suspected [98]. All eligible studies have provided this vital information and were free from any kind of ‘selection bias’. Additionally, evidence-based studies have suggested the potential harmful effects of smoking on the onset and progression of periodontitis, for which smokers were excluded [99,100]. Likewise, the inter-relationship of periodontitis and its systemic manifestations are well-established, resulting in the exclusion of patients with systemic diseases [101,102]. Utilization of ‘placebo/sham’ procedures to enhance clinical outcomes of the trial is an evidence-based verified concept [103]. In the present systematic review, only six out of 31 studies [71,78,92,93,94,96] have utilized a ‘placebo/sham’ procedure as an adjunct to conventional SRP. Furthermore, the role of SRP+aPDT+AB, compared to SRP+AB and SRP+aPDT as well the efficacy of SRP+PS compared to the conventional SRP and SRP+ aPDT have been assessed in this review [83,95]. Differences in study designs were apparent and the majority of clinicians have utilized the SM study design in oral health research [104]. Hence, this meta-analysis included both SM and PG studies in order to assess whether the estimated intervention effect has differed between them. 

### 4.2. Assessment Methods for Various Parameters and Their Inferences to Determine aPDT Efficacy

A decrease in periodontal inflammation is directly proportional to a decrease in the incidence of BOP and detectable plaque levels [105]. Furthermore, the endpoints of a comprehensive periodontal therapy include PPD reduction and CAL gain, both of which are crucial for determining treatment success [106]. Clinical evidence has proven that there is a direct correlation between initial severity of PPD and CAL values and the amount of post-operative differences [107,108,109]. In terms of disease severity at baseline evaluation, a lack of homogeneity in pre-treatment values of PPD and CAL in the data extracted from various studies was observed (Table 1). Therefore, variations in level of significance across clinical parameters were noted, thus making it difficult to provide a cumulative result (Table 5). Utilization of a narrow laser optic fibre tip in deep periodontal pockets facilitates easy and atraumatic periodontal pocket probing ultimately resulting in the PPD and GR reduction post-operatively [3,88]. Furthermore, evidence-based research has proven that clinical outcome remains unaffected by the type of instrumentation utilized in SRP [76,110], which was observed to vary across all eligible studies (Table 1). 

An imbalance amongst the local etiological factors such as dental plaque and calculus, inflammation, and a host defense system can have a great impact on the disease severity and progression [17]. Hence, it is essential to monitor the microbiological and the molecular changes of various growth factors and proinflammatory cytokines. De Oliveira and co-workers have demonstrated, through their studies, the importance of SRP in reducing bacterial load from tooth surface and the failure of aPDT monotherapy in reducing the bacterial counts of *A.a*. periodontal disease activity as well as bone resorption [2,5].

### 4.3. Representation of the Treatment Outcomes

Positive outcomes of any treatment strategy are governed by several factors such as evaluation of disease status at carefully planned follow-up visits, signs of uneventful healing, role of supportive periodontal therapy (SPT) and patient compliance with treatment [111,112,113]. The majority of included studies performed follow-up assessment for up to 3 or 6 months, whereas results of a longer follow-up ranging up to 1–2 years was lacking. Collective data obtained from longitudinal studies have confirmed the role of long-term follow up visits in greater reduction of clinical parameters. These findings have been confirmed by three studies included in the present review [73,77,95]. With regard to healing outcomes, 24 out of 31 studies reported no uneventful healing associated with the absence of any postoperative complications, after application of aPDT [2,3,5,70,71,72,73,74,75,77,78,79,80,81,83,84,85,86,87,88,89,90,92,95]. While six studies have failed to provide any information on healing outcomes [17,76,82,91,93,94], one study [96] has reported a gastrointestinal complication in one patient of the aPDT group. This happens to be unique evidence that has not been registered elsewhere. The presence of residual plaque and calculus resulting in a relapse of periodontal disease severity being inevitable with aPDT monotherapy. Hence, the role of SPT is quintessential. In the present systematic review, apart from the three sequential studies conducted by De Oliveira and co-workers [2,3,5] which utilized aPDT monotherapy, all of the other studies have utilized SRP+aPDT. In the former group of studies, a supragingival professional tooth cleaning was performed 14 days prior to the application of aPDT monotherapy in the treatment sites, which have extensively improved the post-operative clinical findings. Likewise, amongst the studies that have assessed the efficacy of SRP+aPDT, only seven studies [71,75,82,84,90,92,95] have mentioned a planned SPT protocol, which was implemented throughout their study period. Furthermore, information on oral hygiene instructions tailored according to respective study criteria has been specified in all included studies. Co-relation of patient’s compliance in adhering to hygiene instructions cannot be overlooked, since it is vital for treatment success and prevention of disease recurrence [114]. Quantitative measurement of plaque by means various indices can aid in monitoring compliance towards therapy. In connection to this, the assessment of plaque levels was performed in 21 studies included in this review [3,17,71,73,74,75,77,78,79,81,82,84,85,88,89,90,91,92,93,94]. Therefore, an inconsistency in the representation of treatment outcomes in this review is evident.

### 4.4. Role of Laser Parameters

Apart from study methodology, laser parameters are crucial in determining treatment outcome. Calibration of therapeutic power output with a power-meter can aid in regulating low output power for achieving the desired aPDT effect at treatment sites [115]. This technique can also prevent any inadvertent damage caused by utilization of high output power, resulting in a photothermal effect. However, none of the included studies have utilized a power-meter to calibrate the power output. Furthermore, some other parameters that have been overlooked were: emission mode, contact/non-contact mode, energy/treated site, power output, spot size/fibre diameter, fluence and irradiance. Diode lasers have a high affinity to pigment, which, in the case of aPDT, is the PS. However, inflamed periodontal tissues are rich in blood and high levels of proteins, which are also rich in pigments. Traumatic instrumentation can lacerate the sulcus lining and elicit bleeding [23]. Consequently, the overall aPDT effect, which could be achieved by an effective PS dye-laser wavelength combination, will be compromised [23,116]. Additionally, placement of the fibre tip inside the gingival sulcus needs to be performed judiciously, in order to avoid further trauma to inflamed gingival sulcus, serving as a niche for plaque accumulation and favoring disease relapse [3,88].

Bacterial re-colonization after SRP has been proven to occur after three weeks [117] and, hence, multiple aPDT sessions can help to delay this pathological process. Annaji et al., 2016 [46] have compared the efficacy of three sessions (0, 7th and 21st day) versus a single session (0 day) of aPDT in the management of CP, and concluded that the group receiving multiple sessions demonstrated superior treatment outcomes. Nine studies included in this review conducted multiple aPDT sessions [17,71,72,73,74,75,79,90,96], and have unanimously concluded that the utilization of multiple aPDT sessions has positive healing effects. However, the frequency of aPDT application varies in all these studies (Table 2). As a result, a conclusion on the ideal number of sessions and frequency of application of aPDT cannot be drawn.

Additionally, PS concentration and pre-irradiation time (wash-out or PS incubation time) are governed by the binding capacity of PS to target cells [118]. It was observed in 13 out of 31 studies, whereby the PS concentration was not reported [5,70,72,73,74,76,81,84,85,86,91,93,94], whereas a range of PS concentrations have been utilized in the remaining eligible studies. Furthermore, four studies have failed to mention PS pre-irradiation time [5,75,77,90], whereas the remaining 27 studies have reported this time, which ranged from 1–5 min for different PS. An in vitro study by Fumes et al. [119] evaluated the effect of different PS pre-irradiation time periods (1, 2 and 5 min) in aPDT on the biofilms formed by *Streptococcus mutans* and *Candida albicans,* by monitoring the microbial load and have successfully demonstrated that the efficacy of all pre-irradiation times was equal. They emphasized patient discomfort associated with longer pre-irradiation times and thus have advised the use of shorter pre-irradiation times (1 min) in future studies. Up until now, there have been no studies that have determined the minimal duration of PS incubation as well as its role against periopathogens. Hence, further studies on this subject should be sought after. Moreover, discrepancies in the reported data, in terms of the number of sites receiving aPDT application per tooth as well the irradiation time, were noticed amongst the eligible studies (Table 2). These voids have raised concerns regarding the reliability of existing literature, which lacks a reproducible methodology and ultimately hampers the rational use of aPDT in non-surgical management of periodontitis.

### 4.5. Role of RoB Assessment

A vast majority of bias were raised from inadequate randomization, deviations from intended interventions and selective reporting of results (Figure 4 and Figure 5). Another key finding of this systematic review was the presence of a potential conflict of interest in 10 out of the 31 studies [2,3,5,72,73,74,76,79,80,94]. Therefore, the results of the included studies are questionable, and their biased methodology cannot be relied upon. 

### 4.6. Limitations of the Present Systematic Review

The majority of the included studies have assessed the efficacy of SRP+aPDT in comparison to aPDT monotherapy resulting in a lack of meta-analysis on the latter. Utilization of a limited number of teeth (mostly anterior teeth) or on specific sites (interproximal sites in case of deep pockets) or on any two quadrants of the dental arch that could facilitate cross contamination from the untreated sites and overshadow the putative benefits of aPDT was noted. In this review, efficacy of aPDT was monitored in systemically healthy non-smokers only, and its benefits in their immunocompromised counterparts were not established. A lack of long-term follow-up in order to determine the stability in healing after aPDT was also observed. The number of studies included in the meta-analysis was nearly half of the studies eligible for a systematic review due to paucity and inconsistency in available literature. Owing to the aforesaid drawbacks, the objective of this review could not be accomplished.

### 4.7. Future Scope

Future investigations should compare aPDT monotherapy versus SRP+aPDT and provide details of all appropriate laser and PS parameters. Efficacy of aPDT in smokers with various systemic diseases in CP and AgP should be established. The role of patient related outcomes and SPT should be emphasized upon. Nevertheless, future RCTs must have a robust methodology with balanced baseline characteristics, performed by experienced, masked and calibrated clinicians, which will reduce bias. Owing to the evident benefits of a PG-study design, clinicians should prefer its utilization in order to minimize potential ‘carry-over’ effects. Additionally, researchers should conduct a full mouth study protocol with a long-term follow-up assessment of minimum 6 months duration, consisting of, but not limited to, a vast range of clinical, microbiological, radiographic as well as, immune-histological profiles.

## 5. Conclusions

Within the limits of the present systematic review and meta-analysis, it can be concluded that the efficacy of aPDT in improving treatment outcomes, when it is utilized in the non-surgical management of periodontitis, remains debatable. However, the results of a majority of included studies have demonstrated the effectiveness of aPDT, and this role is more pronounced for SRP+aPDT rather than aPDT monotherapy. A careful and critical appraisal was performed which helped to obtain a qualitative assessment of eligible studies, thus highlighting the substantial flaws that prevent a reproducible methodology. Data on standardized aPDT study protocol, ideal PS dye-laser combination, optimal laser and PS parameters remain inconsistent and inconclusive amongst the prevalent literature owing to a highly inferior RoB in many studies. Finally, future research should aim for well-designed, robust and preferably PG-RCTs that will overcome the abovementioned limitations and confounders, in order to achieve palpable progress in this field of research while ensuring the use of an appropriate local laser safety protocol. 

## Figures and Tables

**Table 1:** Tabular representation of eligible in vivo human RCTs in terms of demography, study design, intervention groups, methods of assessment, evaluation period and outcomes. Refer to Supplementary file 2 for list of abbreviations.

Study, Year, Origin and Citation	Journal Name/ Impact Factor (IF)	Study Design	Type of Periodontitis	Sample Size (*n*)	Gender M/F	Age (Years) (Mean ± SD)	Intervention Groups				Evaluation Period	Parameters Assessed	Conclusions
De Oliveira et al., 2009 (Brazil) [2]	Journal of Periodontology IF 2020: 3.742 IF 2009: 2.580	SM-RCT	AgP (A minimum of 20 teeth (mean, 26 teeth) with at least one tooth in each posterior sextant and at least one posterior sextant with a minimum of three natural teeth; ≥5 mm of attachment loss around at least seven teeth involved, excluding first molars and central incisors)	10	2/8	18–35 Mean: 31.01 ± 4.43	SRP (Hand instruments) (10 teeth)		aPDT (10 teeth)		−7 (baseline), 0 (immediately after interventions), +1, +7, +30 and +90 days.	TNF-α and RANKL assessment	NSPT with PDT or SRP led to statistically significant reductions in TNF-a level 30 days following treatment (*p* < 0.05) with no statistically significant intergroup differences (*p* > 0.5).
De Oliveira et al., 2007 (Brazil) [3]	Journal of Periodontology IF 2020: 3.742 IF 2007: 2.426	SM-RCT	AgP (A minimum of 20 teeth (mean, 26 teeth) with at least one tooth in each posterior sextant and at least one posterior sextant with a minimum of three natural teeth; ≥5 mm of attachment loss around at least seven teeth involved, excluding first molars and central incisors)	10	2/8	18–35 Mean: 31.01 ± 4.43	SRP (Hand instruments) (10 teeth)		aPDT (10 teeth)		Baseline, 3 months	PD, RCAL, GR, PI, GI, BOP	PDT and SRP showed statistically significant clinical results (*p* < 0.05) in the non-surgical treatment of aggressive periodontitis with no statistically significant differences (*p* > 0.5) in intergroup comparison.
Novaes et al., 2012 (Brazil) [5]	Lasers in Medical Science IF 2019: 2.574 IF 2012: 2.645	SM-RCT	AgP (A minimum of 20 teeth (mean, 26 teeth) with at least one tooth in each posterior sextant, and at least one posterior sextant with a minimum of three natural teeth; ≥5 mm of attachment loss around at least seven teeth involved, excluding first molars and central incisors)	10	2/8	18–35 Mean: 31	SRP (Hand instruments)		aPDT		−7, 0 (Baseline), and 3 months	Plaque sample analysis for estimation of 40 subgingival species using DNA-DNA hybridization.	aPDT was more effective in reducing the counts of *A.a* (*p* = 0.00) whereas, SRP reduced red complex bacteria. Combination of both treatment methods would be beneficial for the non-surgical treatment of AgP
Franco et al., 2014 (Brazil) [17]	Photodiagnosis and Photodynamic Therapy IF 2020: 2.894 IF 2014: 2.359	SM-RCT	CP (At least 20 teeth with at least one posterior tooth in each quadrant, and periodontal pockets ≥ 5 mm on at least seven teeth)	15	NI	39.5	SRP (Hand instruments)		SRP+aPDT		Baseline and 90 days	BOP, PI, PD, CAL, qPCR gene expression analysis.	Significant improvement in BOP was noted with aPDT group (*p* = 0.03). PDT increased the expression of RANK and OPG, which could indicate a reduction in osteoclastogenesis. Furthermore, the use of PDT in conjunction with conventional treatment significantly increased the expression of FGF2, which has an important role in the periodontal repair process.
Pourabbas et al., 2014 (Iran) [70]	Journal of Periodontology IF 2020: 3.742 IF 2014: 2.900	SM-RCT	CP (≥12 natural teeth with a minimum of three in each quadrant; ≥3 mm attachment loss in about a minimum of 30% of the existing teeth; ≥1 site per quadrant with PPD of ≥4 mm and BOP)	24	10/14	46 ± 8	SRP (Sonic and hand instruments)		SRP+aPDT		Baseline and 3 months	PD, BOP, CAL, GR, IL-1β, TNF-α, MMP-8 and MMP-9 analysis	Intragroup comparison showed significant improvements (*p* < 0.001) for all variables in 3-month follow-up compared with baseline. TNF-α was significantly improved in the SRP+aPDT versus SRP group (*p* < 0.001). Total levels of PMNs were reduced for all patients compared with baseline levels (*p* < 0.001).
Moreira et al., 2015 (Brazil) [71]	Journal of Periodontology IF 2020: 3.742 IF 2015: 3.159	SM-RCT	AgP (A minimum of 20 teeth and two pairs of single rooted contralateral teeth with proximal sites presenting PD and CAL ≥ 5 mm)	20	2/18	18–35 30.6 ± 4.25	SRP + sham procedure (Hand and ultrasonic instruments) 40 teeth/128 sites		SRP+aPDT 40 teeth/135 sites		Baseline,3 months	PD, CAL, GR, PI, BOP Microbiological analysis for counts of 40 bacterial species using DNA- DNA Hybridization Immunological evaluation for GCF levels of IL-1β, IL- 10 and TNF-α.	In deep periodontal pockets analysis (PD ≥ 7 mm at baseline), Test Group presented a decrease in PD and a clinical attachment gain significantly higher than Control Group at 90 days (*p* < 0.05). Test Group also demonstrated significantly less periodontal pathogens of red and orange complexes and a lower ratio IL-1β/IL-10 than Control Group (*p* < 0.05). Four adjunctive sessions of aPDT after SRP have clinical, microbiological and immunological benefits over SRP alone in management of AgP.
Skurska et al., 2015 (Poland) [72]	BMC Oral Health IF 2019: 1.911 IF 2015: 1.605	PG-RCT	AgP (At least 3 sites with PD ≥ 6 mm)	35 SRP+AB: 17 SRP+aPDT:18	12/24 SRP+aPDT: 7/10 SRP+AB: 5/13	23–55 SRP+aPDT: 37.3 ± 8.0 SRP+AB: 34.7 ± 9.0	SRP+ AB 141 sites AB: 375 mg of amoxicillin + 250 mg of metronidazole TDS for 7 days, starting on the day of SRP (Hand and ultrasonic instruments)		SRP+aPDT 137 sites		Baseline, 3 and 6 months	MMP-8 and MMP-9 assessment	In the AB group, patients showed a statistically significant (*p* = 0.01) decrease of MMP-8 GCF level at both 3- and 6-months post treatment. In the PDT group, the change of MMP-8 GCF level was not statistically significant. Both groups showed at 3 and 6 months a decrease in MMP-9 levels. However, this change did not reach statistical significance. SRP+AB is more effective in reducing GCF MMP-8 levels compared to SRP+aPDT.
Arweiler et al., 2014 (Poland) [73]	Clinical Oral Investigations IF 2019: 2.903 IF 2014: 2.704	PG-RCT	AgP (At least 3 sites with PD ≥ 6 mm)	35 SRP+aPDT: 17 SRP+AB: 18	12/24 SRP+aPDT: 7/10 SRP+AB: 5/13	23–55 SRP+aPDT: 37.3 ± 8.0 SRP+AB: 34.7 ± 9.0	SRP+AB 141 sites AB: 375 mg Amoxicillin + 250 mg Metronidazole TDS for 7 days (starting from day of SRP) (Hand and ultrasonic instruments)		SRP+aPDT 137 sites		Baseline, 6 months	PD, CAL, GR, PI, BOP, FMPI, FMBOP	Intragroup comparison revealed statistically significant PD reduction from baseline (*p* < 0.001). SRP+AB showed significant differences in PD reduction and lower number of deep pockets ≥ 7 mm (*p* < 0.001) as compared to SRP+aPDT (*p* = 0.03).
Arweiler et al., 2013 (Poland) [74]	Schweiz Monatsschr Zahnmed IF 2020: NA IF 2013: NA	PG-RCT	AgP (At least 3 sites with PD ≥ 6 mm)	35 SRP+aPDT: 17 SRP+AB: 18	12/24 SRP+aPDT: 7/10 SRP+AB: 5/13	23–55 SRP+aPDT: 37.3 ± 8.0 SRP+AB: 34.7 ± 9.0	SRP+AB 141 sites AB: 375mg Amoxicillin+250 mg MTZ TDS for 7 days (starting from day of SRP) (Hand and ultrasonic instruments)		SRP+aPDT 137 sites		Baseline, 3 months	PD, CAL, GR, PI, BOP, FMPI, FMBOP	SRP+AB showed significant differences in PD reduction, CAL gain and lower number of deep pockets ≥ 7 mm as compared to SRP+aPDT (*p* < 0.001).
Vidal et al., 2017 (Spain) [75]	Journal of Clinical Periodontology IF 2020: 5.241 IF 2017: 4.165	PG-RCT	CP (Four or more periodontal pockets with a PPD ≥ 5 mm and BOP)	37	11/26	55 ± 2	SRP (Hand and ultrasonic instruments)		SRP+aPDT		Baseline, 5, 13 and 25 weeks	PI, PD, GR, CAL, BOP, GCF volume, microbiological and biochemical parameters	RANKL and abundance of *A.a* was significantly decreased in the SRP+aPDT group compared with the SRP group (*p* < 0.05). Except of a reduction in *A.a*, SRP+ aPDT resulted in no additional improvement compared with SRP alone.
Braun et al., 2008 (Germany) [76]	Journal of Clinical Periodontology IF 2020: 5.241 IF 2008: 3.525	SM-RCT	CP (At least one premolar and one molar in every quadrant with a minimum of four teeth each; at least one tooth with an attachment loss of >3 mm in every quadrant)	20	9/11	46.6 ± 6.1	SRP (Hand and piezo- electric ultrasonic instruments)		SRP+aPDT		Baseline, 1 week, 3 months	SFFR, BOP, RAL PD, GR	Values for RAL, PD, SFFR and BOP decreased significantly 3 months after treatment in the control group with a higher impact on the sites treated with adjunctive aPDT (*p* < 0.05). GR increased 3 months after treatment with and without adjunctive aPDT, with no difference between the groups (*p* > 0.05). In patients with CP, clinical outcomes can be improved by adjunctive aPDT.
Berakdar et al., 2012 (Germany) [77]	Head and Face Medicine IF 2020: 1.492 IF 2012: 1.519	SM-RCT	CP (At least four teeth with a PPD of ≥5 mm)	22	12/10	59.3 ± 11.7	SRP (Hand instruments)		SRP+aPDT		Baseline, 1, 3 and 6 months	BOP, PI, PD, CAL	At 1, 3 and 6 months after both types of treatment, an improvement in BOP and CAL was observed. The greater reduction of the PD, achieved by a combination of SRP/PDT, was statistically significant after 6 months (*p* = 0.007).
Raut et al., 2018 (India) [78]	Journal of Indian Society of Periodontology IF 2020: 0.460 IF 2018: 0.44	PG-RCT	CP (PPD > 5 mm and CAL > 4 mm)	50	SRP group: 12/13 SRP+aPDT group: 16/9	SRP group: 46.90 ± 4.32 SRP+aPDT group: 51 ± 2.83	SRP+ sham procedure (Hand and ultrasonic instruments)		SRP+aPDT		Baseline and 6 months	PI, BOP, CAL, PD, microbiological analysis	Significant reduction was seen in PD, CAL and BOP in the test group as compared to control group after 6 months (*p* < 0.05). However, intergroup comparison of PI showed nonsignificant results (*p* > 0.05). Anaerobic culture of plaque samples of test group also revealed a significant reduction of microorganisms in comparison with control group.
Hokari et al., 2018 (Japan) [79]	International Journal of Dentistry IF 2019: 0.58 IF 2018: 0.58	PG-RCT	CP (Moderate: 3–4 mm clinical attachment loss, severe: ≥5 mm loss, generalized: >30% of sites affected)	30	aPDT group: 7/8 MO group: 6/9	aPDT group: 61.4 ± 10.2 MO group: 66.7 ± 9.5	SRP+ Minocycline ointment (MO) (Ultrasonic instruments)		SRP+aPDT		Baseline, 1 and 4 weeks	BOP, PD, CAL, PI, GI, microbiological and inflammatory marker analysis	Local MO administration exhibited a significant decrease in scores for clinical parameters (*p* < 0.01) and a significant reduction in bacterial counts (*p* < 0.01) and IL-1β and IF-γ levels at 1 and 4 weeks after treatment (*p* < 0.01). No significant changes were observed in the aPDT group, except in clinical parameters.
Hill et al., 2019 (Germany) [80]	Photodiagnosis and Photodynamic Therapy IF 2020: 2.894 IF 2019: 2.821	SM-RCT	CP (At least one single and one multi-rooted tooth with at least 4 mm PPD in each quadrant)	20	3/17	61.1	SRP (Hand and piezo- electric ultrasonic instruments)		SRP+aPDT		Baseline, 2 week, 3 and 6 months	BOP, SFFR, PD, GR, RAL, Microbiological analysis	Median values for BOP, RAL, PD, decreased significantly in both groups (*p* < 0.05) after three months of treatment without significant difference between the groups (*p* > 0.05). Two weeks after treatment, the SFFR showed significantly lower mean values in the test group (aPDT). With the applied parameters, this study does not conclusively support ICG-based aPDT, though it is promising because no adverse effects occurred.
Ahad et al., 2016 (India) [81]	Journal of Lasers in Medical Sciences IF 2020: 1.570 IF 2016: 0.68	SM-RCT	CP (At least 2 teeth in different quadrants with PD ≥ 6 mm, and BOP)	30	21/9	38.67 ± 10.52	SRP (Hand and ultrasonic instruments)		SRP+aPDT		Baseline, 1 and 3 months	PI, mSBI, PD, CAL	At 1 month follow-up, intergroup difference in mean change was statistically significant in terms of mSBI and PD for the adjunctive aPDT group (*p* < 0.05), at 3 months interval, no statistically significant difference was observed between test and control groups except in terms of mSBI (*p* > 0.05), thus proving that aPDT improved the gingival status in the nonsurgical management of CP.
Balata et al., 2013 (Brazil) [82]	Journal of Applied Oral Science IF 2019: 2.005 IF 2013: 1.153	SM-RCT	CP (Periodontal pockets with CAL ≥ 5 mm, BOP and radiographic bone loss; minimum of 2 teeth with PD ≥ 7 mm and 2 other teeth with a PD ≥ 5 mm, all with BOP and located on opposite sides of the mouth; and ≥16 teeth in both jaws)	22	8/14	43.18	SRP (Ultrasonic instruments)		SRP+aPDT		Baseline, 1, 3 and 6 months	PI, GI, BOP, GR, CAL	Both groups revealed statistically significant improvement in the clinical parameters (*p* < 0.05) with no statistically significant differences upon intergroup comparison (*p* > 0.05). aPDT did not provide any additional benefit to those obtained with full-mouth ultrasonic debridement used alone.
Bechara et al., 2018 (Brazil) [83]	Photodiagnosis and Photodynamic Therapy IF 2020: 2.894 IF 2018: 2.624	PG-RCT	AgP (Single-rooted teeth in multiple quadrants, with both PPD and CAL ≥ 5 mm, and with BOP)	36 patients (72 sites)	CLM group: 1/17 Placebo group: 1/17	<35 years CLM group: 33.11 ± 4.26 Placebo group: 31.26 ± 4.73	CLM group (*n* = 18) Clarithromycin 500 mg BD for 3 days		Placebo group (*n* = 18)		Baseline, 3 months and 6 months	PD, CAL, BOP, GR	At 3 months, UPD+aPDT, UPD+CLM and UPD + CLM + aPDT groups all exhibited reduced PD relative to the UPD group (*p* < 0.05). However, at 6 months, the mean PD reduction was greater in the antibiotic groups (UPD+CLM and UPD+CLM+aPDT) than in the UPD and UPD+aPDT groups (*p* < 0.05). Regarding clinical attachment level, only the UPD+CLM+aPDT group presented a significant gain relative to the UPD and UPD+aPDT groups (*p* < 0.05).
							UPD + CLM (18 sites)	UPD+ CLM+ aPDT (18 sites)	UPD (18 sites)	UPD+ aPDT (18 sites)			
Bundidpun et al., 2017 (Thailand) [84]	Laser Therapy IF 2020: 0.43 IF 2017: 0.53	SM-RCT	CP (Generalized moderate to severe chronic periodontitis, presence of at least 20 teeth, at least one molar tooth in each quadrant with a minimum of four teeth, at least two teeth and one molar tooth presented with PD > 6 mm in each quadrant)	20	7/13	47.25 ± 8.91	SRP (Piezo-electric ultrasonic instruments)		SRP+aPDT		Baseline, 1, 3 and 6 months	PD, CAL, PI, GBI, GI	All parameters in test group were better than that control group, with statistically significant differences of GBI and GI (*p* < 0.05) at 3 and 6 months after treatment but no statistically significant differences of PD, CAL and PI.
Chitsazi et al., 2014 (Iran) [85]	Journal of Dental Research, Dental Clinics, Dental Prospects IF 2020: 0.69 IF 2014: 1.30	SM-RCT	AgP (Minimum of 12 teeth with at least 3 teeth in each quadrant with ≥4 mm of probing depth)	24	9/15	29	SRP (Piezo-electric ultrasonic instruments)		SRP+aPDT		Baseline, 3 months	PD, CAL, GR, PI, GI, BOP, Microbiological analysis for *A.a*	Intragroup comparison showed an improvement in all the clinical parameters and a significant reduction in the counts of *A.a* at 90 days compared to baseline (*p* < 0.05). None of the periodontal parameters exhibited significant differences between the two groups (*p* > 0.05).
Chitsazi et al., 2014 (Iran) [86]	Journal of Advanced Periodontology and Implant Dentistry IF 2020: NA IF 2014: NA	SM-RCT	CP (At least one site per quadrant exhibiting pocket depth of ≥4 mm with bleeding on probing)	22	10/12	46.1	SRP (Sonic instruments)		SRP+aPDT		Baseline, 1 and 3 months	PD, CAL, BOP, GR, microbiological analysis	PD values decreased significantly in both groups after 1 month (*p* = 0.001) and 3 months (*p* = 0.001) in the SRP and (*p* = 0.001) in the PDT groups the inter-group differences were not significant after 1 (*p* = 0.25) and 3 months (*p* = 0.51). Clinical measurements showed significant decreases after 1 and 3 months at both sites, without inter-group differences, except for BOP after 1 (*p* = 0.004) and 3 months (*p* = 0.0001).
Garcia et al., 2011 (Brazil) [87]	Revista Periodontia IF 2020: NA IF 2011: NA	SM-RCT	AgP (Bone loss first molars and incisors, and other teeth adjacent, with PPD ≥ 5 mm and loss of CAL ≥ 2 mm)	10	4/6	39.3 ± 5.84	SRP (Hand and ultrasonic instruments)		SRP+aPDT		Baseline, 3 months	PD, RCAL, furcation involvement, tooth mobility	Both groups showed improved clinical results in the nonsurgical treatment of AgP with no statistically significant intergroup differences (*p* > 0.05).
Joseph et al., 2014 (India) [88]	Journal of Clinical Periodontology IF 2020: 5.241 IF 2014: 4.641	PG-RCT	CP (A minimum of 20 teeth; PPD 4–6 mm at least in two different quadrants of the mouth)	90	39/51	39.6 ± 8.7	SRP (Hand and ultrasonic instruments)		SRP+aPDT		Baseline, 2 weeks, 1, 3 and 6 months	PPD, CAL, GI, GBI, PI, halitosis.	PD and CAL showed statistically significant reduction in the test group on evaluation at 3 months and 6 months as compared to the control group (*p* < 0.05). A statistically significant improvement in GI and GBI was seen for the test group after 2 weeks and 1 month of aPDT (*p* < 0.01), whereas the improvement in GI and GBI at 3 months and in plaque index at 2 weeks after aPDT was less (*p* < 0.05). In addition, a significant difference was detected for the test group at 1 month in terms of halitosis, which did not persist for long (*p* < 0.05).
Malgikar et al., 2015 (India) [89]	Journal of Dental Lasers IF 2020: 0.696 IF 2015: NA	SM-RCT	CP (At least one site in each quadrant of the mouth having deep PPD ≥ 5 mm and radiographic signs of alveolar bone loss)	24	15/9	M: 36.73 ± 8.46 F: 34.33 ± 6.80	SRP (Hand and piezo- electric ultrasonic instruments)		SRP+aPDT		Baseline, 1, 3 and 6 months.	PI, GI, mSBI, PD, CAL.	A statistically significant decrease in PD, CAL, PI, GI, mSBI scores was seen in SRP+aPDT at the end of 6 months (*p* < 0.001).
Monzavi et al., 2016 (Iran) [90]	Photodiagnosis and Photodynamic Therapy IF 2020: 2.894 IF 2016: 2.503	SM-RCT	CP (At least three teeth exhibiting residual pocket depth of ≥ 5 mm with bleeding on probing)	50	25/25	49.6 ± 8.5	SRP (Hand and ultrasonic instruments)		SRP+aPDT		Baseline, 1 and 3 months	BOP, PI, CAL, PPD, FMPS, FMBS	There were no significant differences between two groups at baseline. BOP, PPD and FMBS showed significant improvements in the test group (*p* ≤ 0.001). In terms of PI, FMPS and CAL, no significant differences were observed between both groups (*p* ≥ 0.05).
Raj et al., 2016 (India) [91]	Indian Journal of Dental Research IF 2020: 0.37 IF 2016: 0.08	PG-RCT	CP (More than 16 natural teeth; PPD ≥ 5 mm)	20	8/12	NI	SRP (Type of instruments utilized-NI)		SRP+aPDT		Baseline and 3 months	PI, GI, PD, CAL and microbiological analysis	There was a significant reduction in PI, GI, PD, CAL and microbiologic parameters in test group, following SRP and PDT, when compared with SRP alone in control group (*p* < 0.001). SRP+aPDT has shown additional improvement in periodontal parameters when compared to SRP alone and has a beneficial effect in chronic periodontitis patients.
Sena et al., 2019 (Brazil) [92]	Photobiomodulation, Photomedicine and Laser Surgery IF 2019: 1.913	SM-RCT	CP (At least six sites with PD 5–9 mm; and BOP)	9 (6 sites/ patient: total-54 sites)	NI	NI	SRP+ placebo procedure (Hand and ultrasonic instruments)		SRP+aPDT		Baseline and 3 months	BOP, PD, CAL, VPI	There was a statistically significant decrease in BOP for test group (*p* = 0.003) and control group (*p* = 0.001). Intragroup comparison for PD and CAL showed statistically significant differences from baseline (*p* < 0.05) with no intergroup differences (*p* > 0.05). Hence, SRP+aPDT did not show any additional benefits over SRP alone.
Shingnapurkar et al., 2016 (India) [93]	Indian Journal of Dental Research IF 2020: 0.37 IF 2016: 0.08	SM-RCT	CP (PD > 5 mm)	60 sites	NI	NI	SRP+ sham procedure (Hand and ultrasonic instruments)		SRP+aPDT		Baseline, 1 and 3 months	PI, GI, PD, RAL	Mean baseline values for PI, GI, PPD and RAL were not different in the test group and control group. Statistically significant difference in PPD and RAL, 3 months after treatment was seen in test group as compared to the control group (*p* < 0.05).
Sigusch et al., 2010 (Germany) [94]	Journal of Periodontology IF 2020: 3.742 IF 2010: 2.946	PG-RCT	CP (<30% of sites with PPD >3.5 mm)	24 (12 in each group)	PDT group: 4/8 Control group: 3/9	PDT group F: 39.75 M: 45 Control group: F: 44.22 M:42.67	SRP+ sham procedure (Type of instruments utilized- NI)		SRP+aPDT		Baseline, 1, 4 and 12 weeks.	PI, reddening, PD, BOP, CAL, GR Quantitative analysis for *F.n*.	In patients with localized CP who received aPDT treatment, significant reductions in reddening, BOP, and mean PD and CAL were observed during the observation period and with respect to controls (*p* < 0.001). Four and 12 weeks after aPDT, the mean PD and CAL showed significant differences from baseline values and from those of the control group. In the aPDT group, 12 weeks after treatment, the *F.n.* DNA concentration was found to be significantly reduced compared to the baseline level (*p* < 0.001) compared to control group.
Theodoro et al., 2012 (Brazil) [95]	Lasers in Medical Science IF 2019: 2.574 IF 2012: 2.645	SM-RCT	CP (At least three non-adjacent sites with BOP and a PD of 5–9 mm at least 20 teeth in the oral cavity)	33	12/21	43.12 ± 8.2	SRP (Hand instruments)		SRP+ PS (TBO) only	SRP+aPDT	Baseline, 60, 90 and 180 days	VPI, GI, BOP, PD, CAL, GR, microbiological analysis	All treatment groups showed an improvement in all clinical parameters, and a significant reduction in the proportion of sites positive for periodontopathogens at 60, 90 and 180 days compared to baseline (*p* < 0.05). None of the periodontal parameters showed a significant difference among the groups (*p* > 0.05). At 180 days, PDT treatment led to a significant reduction in the percentage of sites positive for all bacteria compared to SRP alone (*p* < 0.05).
Theodoro et al., 2017 (Brazil) [96]	Journal of Photochemistry and Photobiology B IF 2020: 4.383 IF 2017: 3.438	PG-RCT	CP (Severe generalized CP in at least 6 teeth and with one or several sites with PD ≥ 5 mm; a loss of CAL ≥ 5 mm; a minimum of 30% of the sites with PD and CAL ≥ 4 mm and BOP; and the presence of at least 15 teeth)	34	AB group: 7/7 aPDT group: 9/5	AB group: 46.3 ± 6.8 aPDT group: 48.8 ± 8.3	SRP+ (MTZ+ AMX) MTZ dose: 400mg TDS-7 days AMX dose: 500mg TDS-7 days (Type of instruments utilized for SRP-NI)		SRP +aPDT+ placebo pills		Baseline and 90 days	BOP, PD, CAL	There was a significant improvement in CAL only in the intermediate pocket in the aPDT group com- pared to the MTZ + AMX group between baseline and 90 days post-treatment (*p* = 0.01). There was a reduction of both BOP and the percentage of residual pockets at 90 days after treatment compared with baseline in both groups (*p* < 0.05).

**Table 2:** Tabular representation of PS dye and laser parameters utilized for aPDT in the selected eligible in vivo human studies. Refer to Supplementary file 2 for list of abbreviations.

Study, Year, Origin and Citation	Photosensitizer (PS) Used and Its Concentration	Pre-Irradiation Exposure Time to PS (min)	Laser Wavelength Utilized	Emission Mode Contact/No Contact Tip Initiation	Energy (J)	Power Output (W)	Pulse Length (Duration), Pulse Interval	Use of Power Meter	Distance from Target	Spot Size/Fibre-Tip Diameter/Spot Diameter	Energy Density [Fluence] (J/cm^2^)	Power Density [Irradiance] (W/cm^2^)	Exposure Time to Laser Irradiation [Minute (min)/Second (s)]	No. of aPDT Applications
De Oliveira et al., 2009 (Brazil) [2]	Phenothiazine chloride (10 mg/mL)	1 min	660 nm	Contact mode, fibre tip was place at the entrance of the gingival sulcus	NI	NI	NI	NI	NA	Tip diameter: 600 µm	NI	60 mW/cm^2^	10 s/site (6 sites = 1 min/tooth)	1
De Oliveira et al., 2007 (Brazil) [3]	Phenothiazine chloride (10 mg/mL)	1 min	660 nm	Contact mode, fibre tip was place at the entrance of the gingival sulcus	NI	NI	NI	NI	NI	Tip diameter: 600 µm	NI	60 mW/cm^2^	10 s/site (6 sites = 1 min/tooth)	1
Novaes et al., 2012 (Brazil) [5]	Phenothiazine chloride	NI	660 nm	Contact mode, fibre tip was place at the entrance of the gingival sulcus	NI	NI	NI	NI	NI	Tip diameter: 600 µm [8.5 cm long optic fibre with 60° angulated tip] Spot size: 0.06	212.23 J/cm^2^	60 mW/cm^2^	10 s/site (6 sites/tooth) 60 s/tooth	1
Franco et al., 2014 (Brazil) [17]	Methylene blue (0.01%)	5 min	660 nm	NI	NI	NI	NI	NI	NI	NI	5.4 J/cm^2^	60 mW/cm^2^	5 s/site (6 sites/tooth) 90 s/tooth	4
Pourabbas et al., 2014 (Iran) [70]	Toluidine blue	60 s	638 nm	NI	NI	NI	NI	NI	NI	NI	8–10 J/cm^2^	NI	120 s	1
Moreira et al., 2015 (Brazil) [71]	Phenothiazine chloride (10 mg/mL)	1 min	670 nm	NI	NI	75 mW	NI	NI	NI	Tip diameter: 600 µm	Fluence/site: 2.49 J/cm^2^ Fluence/tooth: 14.94 J/cm^2^	0.25 W/cm^2^	10 s /site	4 (0, 2nd, 7th and 14th day)
Skurska et al., 2015 (Poland) [72]	Phenothiazine chloride	3 min	660 nm	NI	NI	NI	NI	NI	NI	NI	120 J/cm^2^	60 mw/cm^2^	60 s/site	2 (0 and 7th day)
Arweiler et al., 2014 (Poland) [73]	Phenothiazine chloride	3 min	660 nm	NI	NI	NI	NI	NI	NI	NI	120 J/cm^2^	60 mw/cm^2^	60 s/site	2 (0 and 7th day)
Arweiler et al., 2013 (Poland) [74]	Phenothiazine chloride	3 min	660 nm	NI	NI	NI	NI	NI	NI	NI	120 J/cm^2^	60 mw/cm^2^	60 s/site	2 (0 and 7th day)
Vidal et al., 2017 (Spain) [75]	Methylene blue (0.005%)	NI	670 nm	NI	NI	150 mW	NI	NI	NI	NI	NI	NI	60 s/pocket	3 (1, 5 and 13 weeks)
Braun et al., 2008 (Germany) [76]	Phenothiazine chloride	3 min	660 nm	NI	NI	100 mW	NI	NI	NI	NI	NI	NI	10 s/site (6 sites = 1 min /tooth)	1
Berakdar et al., 2012 (Germany) [77]	Methylene blue 0.005%	NI	670 nm	NI	NI	150 mW	NI	NI	NI	NI	NI	NI	1 min	1
Raut et al., 2018 (India) [78]	Indocyanine green (5 mg/mL)	60 s	810 nm	CW, contact mode	NI	80 mW	NI	NI	NA	NI	5.4 J/cm^2^	NI	60 s	1
Hokari et al., 2018 (Japan) [79]	Methylene blue dye 0.01%	1 min	670 nm	NI, contact mode	NI	140 mW	NI	NI	NA	NI	21 J/cm^2^	NI	60 s	2 (0 and 7th day)
Hill et al., 2019 (Germany) [80]	Indocyanine green (0.1 mg/mL)	60 s	808 nm	NI	NI	100 mW	NI	NI	NI	Tip diameter: 300 µm	2829 J/cm^2^	NI	NI	1
Ahad et al., 2016 (India) [81]	Phenothiazine chloride	3 min	660 nm	Contact mode	NI	NI	NI	NI	NA	Tip diameter: 0.6 µm	NI	100 mW/cm^2^	10 s/site (6 sites, 1 min/tooth)	1
Balata et al., 2013 (Brazil) [82]	Methylene blue 0.005%	2 min	660 nm	90° angle with the gingival surface and with no contact with the tissues	9 J	100 mW	NI	NI	NI	Tip diameter: 600 µm tip	320 J/cm^2^	NI	90 s/site	1
Bechara et al., 2018 (Brazil) [83]	Methylene Blue (10 mg/mL)	1 min	660 nm	NI	NI	60 mW	NI	NI	NI	NI	129 J/cm^2^	NI	60 s/tooth (2 sites/tooth)	1
Bundidpun et al., 2017 (Thailand) [84]	Phenothiazine chloride	1 min	660 nm	Contact mode	NI	100 mW	NI	NI	NA	NI	NI	NI	10 s/site (6 sites) 1 min/tooth	1
Chitsazi et al., 2014 (Iran) [85]	Toluidine Blue	1 min	670–690 nm	Contact mode	NI	75 mW	NI	NI	NA	NI	NI	NI	120 s/site	1
Chitsazi et al., 2014 (Iran) [86]	Tolonium chloride (Toluidine Blue O)	60 s	638 nm	Contact mode	NI	NI	NI	NI	NA	NI	8–10 J/cm^2^	NI	120 s	1
Garcia et al., 2011 (Brazil) [87]	Methylene blue (0.005%)	5 min	660 nm	NI	NI	40 mW	NI	NI	NI	NI	120 J/cm^2^	NI	120 s/site	1
Joseph et al., 2014 (India) [88]	Methylene blue (10 mg/mL)	3 min	655 nm	CW, contact mode, tip was inserted into the gingival sulcus	NI	NI	NI	NI	NA	Tip diameter: 200 µm Probe tip diameter: 0.5 mm	NI	60 mW/ cm^2^	60 s/site (4 sites/ tooth)	1
Malgikar et al., 2015 (India) [89]	Methylene blue 1%	3 min	980 nm	Contact mode, tip was initiated	NI	Peak Power: 5 W Average power 1 W	Pulse length: 200 µs, Pulse interval: 200	NI	NA	Tip diameter: 400 µm	NI	NI	30–45 s/site	1
Monzavi et al., 2016 (Iran) [90]	Indocyanine green (1 mg/mL)	NI	810 nm	CW, contact mode	PBM tip: 6 J Bulb tip: 4 J	200 mW	NI	NI	NA	Use of two types of tips: PBM tip was placed on papilla and then the bulb tip was inserted inside the pocket from each buccal or lingual/palatal side, moving from the bottom of the pocket to the coronal aspect.	NI	NI	PBM tip: 30 s Bulb tip: 10 s	4 (0, 7th, 17th and 27th days)
Raj et al., 2016 (India) [91]	Toluidine blue	1 min	635 nm	Contact mode	NI	500 W	NI	NI	NA	NI	NI	NI	60 s	1
Sena et al., 2019 (Brazil) [92]	Chloro-aluminum pthalocyanine (AlClFc) 5 µM	5 min	660 nm	CW, laser optical fiber tip was positioned parallel to the tooth axis in contact with the gingival margin (without penetrating the pocket)	1.5 J	100 mW	NI	NI	NA	Spot size: 0.028 cm^2^	54 J/cm^2^	4 W/cm^2^	15 s	1
Shingnapurkar et al., 2016 (India) [93]	Indocyanine green (1 mg/mL)	3 min	810 nm	Gated CW, Contact mode	3 J	200 mW	Pulse duration: 25 µm Duty cycle 50%	NI	NA	Tip diameter: 400 µm	0.0125 J/cm^2^	NI	30 s/site	1
Sigusch et al., 2010 (Germany) [94]	Phenothiazine chloride	1 min	660 nm	Contact mode	NI	NI	NI	NI	NA	Tip diameter: 600 µm tip	NI	60 mW/cm^2^	10 s/site (6 sites =1 min /tooth)	1
Theodoro et al., 2012 (Brazil) [95]	Toluidine blue O 100 µg/mL	1 min	660 nm	The laser optical fiber tip was positioned parallel to and in contact with the selected site	4.5 J	30 mW	NI	NI	NA	Spot size: 0.07 cm^2^	64.28 J/cm^2^	0.4 W/cm^2^	150 s	1
Theodoro et al., 2017 (Brazil) [96]	Methylene blue (10 mg/mL)	1 min	660 nm	Contact mode	4.8 J	100 mW	NI	NI	NA	Spot size 0.03 cm^2^	160 J/cm^2^	NI	48 s	3 (0, 48 h, 96 h)

**Table 3:** Forest plots illustrating the overall PPD reduction and CAL gain at three months. Refer to Supplementary file 2 for a list of abbreviations.

**Overall PPD Reduction for SM Studies at 3 Months**						
Study	SMD	SE	95% CI	Weight (%)	*P* = 0.463	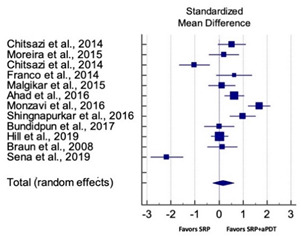
Chitsazi et al., 2014	0.525	0.289	−0.056 to 1.106	8.28		
Moreira et al., 2015	0.205	0.311	−0.425 to 0.834	8.11		
Chitsazi et al., 2014	−1.023	0.316	−1.659 to −0.386	8.07		
Franco et al., 2014	0.631	0.364	−0.115 to 1.378	7.67		
Malgikar et al., 2015	0.119	0.284	−0.453 to 0.691	8.31		
Ahad et al., 2016	0.639	0.204	0.235 to 1.043	8.88		
Monzavi et al., 2016	1.669	0.231	1.211 to 2.127	8.70		
Shingnapurkar et al., 2016	0.995	0.271	0.454 to 1.537	8.42		
Bundidpun et al., 2017	0.007	0.310	−0.620 to 0.635	8.11		
Hill et al., 2019	0.039	0.072	−0.103 to 0.181	9.47		
Braun et al., 2008	0.139	0.310	−0.490 to 0.767	8.11		
Sena et al., 2019	−2.169	0.340	−2.851 to −1.487	7.87		
Total (random effects)	0.166	0.227	−0.278 to 0.611	100.00		
Heterogeneity: Q = 15.81; DF = 11; *P* = 0.0001; I^2^ = 91.21%						
**Overall PPD Reduction for PG Studies at 3 Months**						
Study	SMD	SE	95% CI	Weight (%)	*P* = 0.763	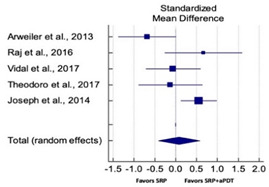
Arweiler et al., 2013	−0.681	0.340	−1.374 to 0.011	19.80		
Raj et al., 2016	0.669	0.441	−0.258 to 1.595	15.89		
Vidal et al., 2017	−0.060	0.322	−0.714 to 0.593	20.59		
Theodoro et al., 2017	−0.127	0.374	−0.897 to 0.643	18.43		
Joseph et al., 2014	0.556	0.215	0.127 to 0.984	25.28		
Total (random effects)	0.076	0.252	−0.420 to 0.573	100.00		
Heterogeneity: Q = 11.87; DF = 4; *P* = 0.018; I^2^ = 66.31%						
**Overall CAL Gain for SM Studies at 3 Months**						
Study	SMD	SE	95% CI	Weight (%)	*P* = 0.088	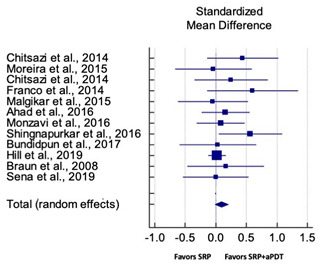
Chitsazi et al., 2014	0.439	0.287	−0.140 to 1.017	3.52		
Moreira et al., 2015	−0.040	0.310	−0.667 to 0.588	3.02		
Chitsazi et al., 2014	0.249	0.297	−0.351 to 0.849	3.29		
Franco et al., 2014	0.601	0.364	−0.144 to 1.346	2.20		
Malgikar et al., 2015	−0.048	0.284	−0.620 to 0.523	3.60		
Ahad et al., 2016	0.158	0.199	−0.237 to 0.552	7.35		
Monzavi et al., 2016	0.080	0.199	−0.314 to 0.474	7.37		
Shingnapurkar et al., 2016	0.564	0.260	0.043 to 1.084	4.30		
Bundidpun et al., 2017	0.032	0.310	−0.595 to 0.660	3.02		
Hill et al., 2019	0.019	0.072	−0.123 to 0.161	55.29		
Braun et al., 2008	0.161	0.310	−0.468 to 0.789	3.01		
Sena et al., 2019	0.000	0.268	−0.538 to 0.538	4.04		
Total (random effects)	0.092	0.233	−0.013 to 0.198	100.00		
Heterogeneity: Q = 8.74; DF = 11; *P* = 0.655; I^2^ = 0.00%						
**Overall CAL Gain for PG Studies at 3 Months**						
Study	SMD	SE	95% CI	Weight (%)	*P* = 0.745	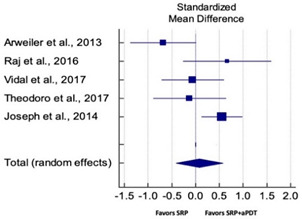
Arweiler et al., 2013	−0.662	0.340	−1.356 to 0.010	19.80		
Raj et al., 2016	0.669	0.441	−0.258 to 1.595	15.89		
Vidal et al., 2017	−0.102	0.372	−0.514 to 0.793	20.59		
Theodoro et al., 2017	−0.106	0.374	−0.997 to 0.743	18.43		
Joseph et al., 2014	0.456	0.255	−0.120 to 0.673	25.28		
Total (random effects)	0.056	0.358	−0.408 to 0.552	100.00		
Heterogeneity: Q = 8.95; DF = 4; *P* = 0.028; I^2^ = 70.31%						

**Table 4:** Forest plots illustrating the overall PPD reduction and CAL gain at 6 months. Refer to Supplementary file 2 for a list of abbreviations.

**Overall PPD Reduction for SM Studies at 6 Months**						
Study	SMD	SE	95% CI	Weight (%)	*P* = 0.935	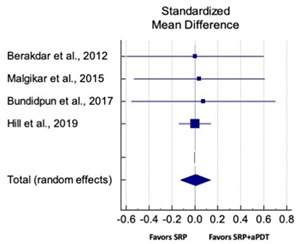
Berakdar et al., 2012	0.040	0.296	−0.598 to 0.598	5.08		
Malgikar et al., 2015	0.037	0.284	−0.535 to 0.609	5.52		
Bundidpun et al., 2017	0.072	0.310	−0.555 to 0.701	4.63		
Hill et al., 2019	0.060	0.072	−0.142 to 0.142	84.77		
Total (random effects)	0.005	0.066	−0.126 to 0.136	100.00		
Heterogeneity: Q = 0.06; DF = 3; *P* = 0.99; I^2^ = 0.00%						
**Overall PPD Reduction for PG Studies at 6 Months**						
Study	SMD	SE	95% CI	Weight (%)	*P* = 0.809	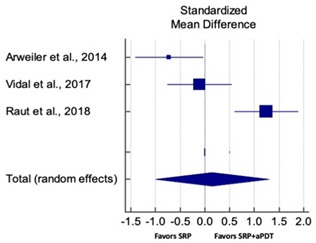
Arweiler et al., 2014	−0.722	0.342	−1.417 to −0.027	33.04		
Vidal et al., 2017	−0.109	0.322	−0.763 to 0.545	33.47		
Raut et al., 2018	1.241	0.321	0.594 to 1.888	33.49		
Total (random effects)	0.141	0.579	−1.007 to 1.288	100.00		
Heterogeneity: Q = 18.71; DF = 2; *P* = 0.0001; I^2^ = 89.31%						
**Overall CAL Gain for SM Studies at 6 Months**						
Study	SMD	SE	95% CI	Weight (%)	*P* = 0.846	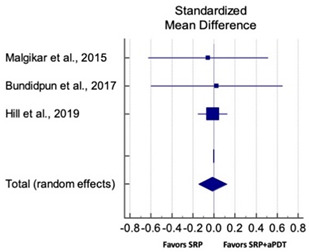
Malgikar et al., 2015	−0.057	0.284	−0.629 to 0.515	5.82		
Bundidpun et al., 2017	0.025	0.310	−0.602 to 0.653	4.88		
Hill et al., 2019	−0.012	0.072	−0.155 to 0.130	89.30		
Total (random effects)	−0.013	0.068	−0.148 to 0.121	100.00		
Heterogeneity: Q = 0.03; DF = 2; *P* = 0.984; I^2^ = 0.00%						
**Overall CAL Gain for PG Studies at 6 Months**						
Study	SMD	SE	95% CI	Weight (%)	*P* = 0.018	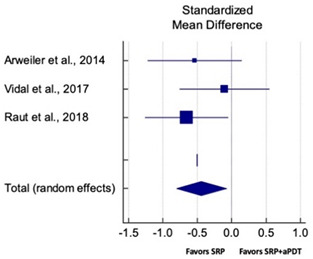
Arweiler et al., 2014	−0.539	0.337	−1.224 to 0.146	29.91		
Vidal et al., 2017	−0.103	0.322	−0.756 to 0.551	32.69		
Raut et al., 2018	−0.658	0.301	−1.265 to −0.050	37.40		
Total (random effects)	−0.441	0.184	−0.805 to −0.075	100.00		
Heterogeneity: Q = 1.70; DF = 2; *P* = 0.42; I^2^ = 0.00%						

**Table 5:** Tabular representation describing the assessment of clinical parameters used for the selected eligible in vivo human studies. Refer to Supplementary file 2 for a list of abbreviations.

Study, Year, Origin and Citation	PPD		CAL		BOP/SBI		PI		GI		GR	
	Statistically Significant Y/N/NI/NS	Not Statistically Significant Y/N/NI/NS	Statistically Significant Y/N/NI/NS	Not Statistically Significant Y/N/NI/NS	Statistically Significant Y/N/NI/NS	Not Statistically Significant Y/N/NI/NS	Statistically Significant Y/N/NI/NS	Not Statistically Significant Y/N/NI/NS	Statistically Significant Y/N/NI/NS	Not Statistically Significant Y/N/NI/NS	Statistically Significant Y/N/NI/NS	Not Statistically Significant Y/N/NI/NS
De Oliveira et al., 2007 (Brazil) [3]	N	Y	N	Y	N	Y	N	Y	N	Y	N	Y
Franco et al., 2014 (Brazil) [17]	N	Y	N	Y	Y	N	N	Y	NS	NS	NS	NS
Pourabbas et al., 2014 (Iran) [70]	N	Y	N	Y	N	Y	NS	NS	NS	NS	N	Y
Moreira et al., 2015 (Brazil) [71]	Y	N	Y	N	Y	N	Y	N	NS	NS	Y	N
Arweiler et al., 2014 (Poland) [73]	N	Y	N	Y	N	Y	N	Y	NS	NS	N	Y
Arweiler et al., 2013 (Poland) [74]	N	Y	N	Y	N	Y	N	Y	NS	NS	N	Y
Vidal et al., 2017 (Spain) [75]	N	Y	N	Y	N	Y	N	Y	NS	NS	N	Y
Braun et al., 2008 (Germany) [76]	Y	N	Y	N	Y	N	NS	NS	NS	NS	N	Y
Berakdar et al., 2012 (Germany) [77]	Y	N	Y	N	N	Y	N	Y	NS	NS	NS	NS
Raut et al., 2018 (India) [78]	Y	N	Y	N	Y	N	N	Y	NS	NS	NS	NS
Hokari et al., 2018 (Japan) [79]	N	Y	N	Y	N	Y	N	Y	N	Y	NS	NS
Hill et al., 2019 (Germany) [80]	N	Y	N	Y	N	Y	NS	NS	NS	NS	N	Y
Ahad et al., 2016 (India) [81]	N	Y	N	Y	Y	N	N	Y	NS	NS	NS	NS
Balata et al., 2013 (Brazil) [82]	N	Y	N	Y	N	Y	N	Y	N	Y	N	Y
Bechara et al., 2018 (Brazil) [83]	Y	N	Y	N	Y	N	NS	NS	NS	NS	Y	N
Bundidpun et al., 2017 (Thailand) [84]	N	Y	N	Y	Y	N	N	Y	Y	N	NS	NS
Chitsazi et al., 2014 (Iran) [85]	N	Y	N	Y	N	Y	N	Y	N	Y	N	Y
Chitsazi et al., 2014 (Iran) [86]	N	Y	N	Y	N	Y	NS	NS	NS	NS	N	Y
Garcia et al., 2011 (Brazil) [87]	N	Y	N	Y	NS	NS	NS	NS	NS	NS	NS	NS
Joseph et al., 2014 (India) [88]	Y	N	Y	N	N	Y	N	Y	N	Y	NS	NS
Malgikar et al., 2015 (India) [89]	Y	N	Y	N	Y	N	Y	N	Y	N	NS	NS
Monzavi et al., 2016 (Iran) [90]	Y	N	N	Y	Y	N	N	Y	NS	NS	NS	NS
Raj et al., 2016 (India) [91]	Y	N	N	Y	NS	NS	Y	N	Y	N	NS	NS
Sena et al., 2019 (Brazil) [92]	N	Y	N	Y	N	Y	N	Y	NS	NS	NS	NS
Shingnapurkar et al., 2016 (India) [93]	Y	N	Y	N	NS	NS	N	Y	N	Y	NS	NS
Sigusch et al., 2010 (Germany) [94]	Y	N	Y	N	Y	N	Y	N	NS	NS	Y	N
Theodoro et al., 2012 (Brazil) [95]	N	Y	N	Y	N	Y	N	Y	N	Y	N	Y
Theodoro et al., 2017 (Brazil) [96]	N	Y	N	Y	N	Y	NS	NS	NS	NS	NS	NS

**Table 6:** Forest plots based on sensitivity analysis illustrating the overall PPD reduction and CAL gain at 3 months without outlier studies. Refer to Supplementary file 2 for a list of abbreviations.

**Overall PPD Reduction for SM Studies at 3 Months**						
Study	SMD	SE	95% CI	Weight (%)	*P* = 0.153	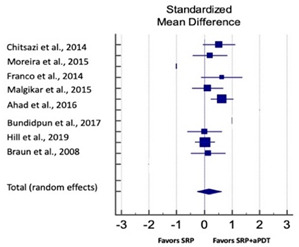
Chitsazi et al., 2014	0.525	0.289	−0.056 to 1.106	12.28		
Moreira et al., 2015	0.205	0.311	−0.425 to 0.834	12.11		
Franco et al., 2014	0.631	0.364	−0.115 to 1.378	11.67		
Malgikar et al., 2015	0.119	0.284	−0.453 to 0.691	12.31		
Ahad et al., 2016	0.639	0.204	0.235 to 1.043	12.88		
Bundidpun et al., 2017	0.007	0.310	−0.620 to 0.635	12.11		
Hill et al., 2019	0.039	0.072	−0.103 to 0.181	14.47		
Braun et al., 2008	0.139	0.310	−0.490 to 0.767	12.11		
Total (random effects)	0.282	0.234	−0.286 to 0.624	100.00		
Heterogeneity: Q = 9.14; DF = 7; *P* = 0.71; I^2^ = 0.00%						
**Overall PPD Reduction for PG Studies at 3 Months**						
Study	SMD	SE	95% CI	Weight (%)	*P* = 0.361	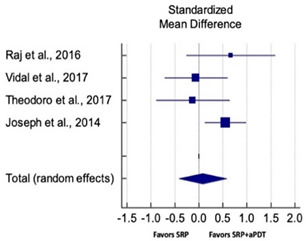
Raj et al., 2016	0.669	0.441	−0.258 to 1.595	18.89		
Vidal et al., 2017	−0.060	0.322	−0.714 to 0.593	25.59		
Theodoro et al., 2017	−0.127	0.374	−0.897 to 0.643	22.43		
Joseph et al., 2014	0.556	0.215	0.127 to 0.984	33.19		
Total (random effects)	0.257	0.278	−0.230 to 0.683	100.00		
Heterogeneity: Q = 8.87; DF = 3; *P* = 0.22; I^2^ = 0.00%						
**Overall CAL Gain for SM Studies at 3 Months**						
Study	SMD	SE	95% CI	Weight (%)	*P* = 0.166	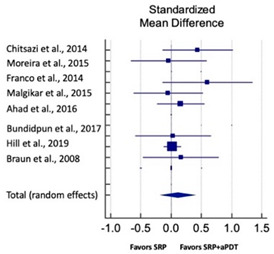
Chitsazi et al., 2014	0.439	0.287	−0.140 to 1.017	5.52		
Moreira et al., 2015	−0.040	0.310	−0.667 to 0.588	5.02		
Franco et al., 2014	0.601	0.364	−0.144 to 1.346	4.20		
Malgikar et al., 2015	−0.048	0.284	−0.620 to 0.523	5.60		
Ahad et al., 2016	0.158	0.199	−0.237 to 0.552	10.35		
Bundidpun et al., 2017	0.032	0.310	−0.595 to 0.660	5.02		
Hill et al., 2019	0.019	0.072	−0.123 to 0.161	59.29		
Braun et al., 2008	0.161	0.310	−0.468 to 0.789	5.01		
Total (random effects)	0.162	0.253	−0.326 to 0.406	100.00		
Heterogeneity: Q = 8.40; DF = 7; *P* = 0.625; I^2^ = 0.00%						
**Overall CAL Gain for PG Studies at 3 Months**						
Study	SMD	SE	95% CI	Weight (%)	*P* = 0.234	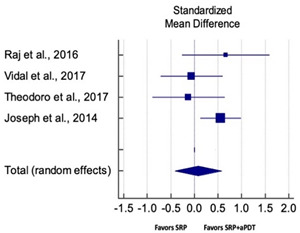
Raj et al., 2016	0.669	0.441	−0.258 to 1.595	18.89		
Vidal et al., 2017	−0.102	0.372	−0.514 to 0.793	25.59		
Theodoro et al., 2017	−0.106	0.374	−0.997 to 0.743	22.43		
Joseph et al., 2014	0.456	0.255	−0.120 to 0.673	33.19		
Total (random effects)	0.227	0.352	−0.420 to 0.673	100.00		
Heterogeneity: Q = 9.7; DF = 3; *P* = 0.22; I^2^ = 0.00%						

## Supplementary Materials

The following are available online at https://www.mdpi.com/article/10.3390/pharmaceutics13060836/s1, PRISMA Checklist (Supplementary file 1), List of abbreviations (Supplementary file 2).
