# Supplementary material for: Effectiveness of Antimicrobial Photodynamic Therapy in the Treatment of Periodontitis: A Systematic Review and Meta-Analysis of In Vivo Human Randomized Controlled Clinical Trials
Source: Pharmaceutics. 2021 Jun 4;13(6):836. doi: 10.3390/pharmaceutics13060836 (PMC8228221; doi:10.3390/pharmaceutics13060836)
Supplement: Supplementary file 1 [file pharmaceutics-13-00836-s001.zip › Supplementary file 2.pdf]

pharmaceutics

Supplementary Materials: Effectiveness of Antimicrobial Photodynamic Therapy in the Treatment of Periodontitis: A Systematic Review and Meta-Analysis of In Vivo Human Randomized Controlled Clinical Trials

Snehal Dalvi, Stefano Benedicenti, Tudor Sălăgean, Ioana Roxana Bordea and Reem Hanna

List of abbreviations [In alphabetical order]

Scheme .	Abbreviation	Full Form
1.	aPDT	Antimicrobial Photodynamic Therapy
2.	A.a	Aggregatibacter actinomycetemcomitans
3.	AgP	Aggressive Periodontitis
4.	AAP	American Academy of Periodontology
5.	AB	Systemic or local antibiotics
6.	BD	Twice daily
7.	BOP	Bleeding on probing
8.	COX-2	Cyclooxygenase- 2
9.	CAL	Clinical attachment level
10.	CCRCT	Cochrane Central Register of Controlled Trials
11.	CLM	Clarithromycin
12.	CP	Chronic periodontitis
13.	CRP	C-reactive protein.
14.	CI	Confidence interval
15.	cm	Centimeter
16.	cm^2	Square centimeter
17.	DF	Degrees of freedom
18.	ELISA	Enzyme-linked immunosorbent assay
19.	et al	And others
20.	FGF-2	Fibroblast growth factor 2
21.	FMPI	Full mouth plaque index
22.	FMBOP	Full mouth bleeding on probing
23.	F.n.	Fusobacterium nucleatum
24.	GI	Gingival index
25.	GBI	Gingival bleeding index
26.	GCF	Gingival crevicular fluid
27.	GR	Gingival recession
28.	IL- 1β	Interleukin-1 beta
29.	IF-γ	Interferon- gamma

30.	IF	Impact Factor
31.	I^2	Statistical test for percentage variation due to heterogeneity
32.	J	Joule
33.	J/cm^2	Joules per square centimeter
34.	LLLT	Low level laser therapy
35.	MB	Methylene blue
36.	MeSH	Medical Subject Headings
37.	MMP-8	Matrix metalloproteinase-8
38.	MMP- 9	Matrix metalloproteinase-9
39.	mSBI	Modified sulcus bleeding index
40.	min	Minute
41.	μm	Micrometer
42.	mm	Millimeter
43.	mW	Milliwatt
44.	M/F	Male/ Female
45.	MTZ	Metronidazole
46.	n	Sample size
47.	N	No
48.	nm	Nanometer
49.	NSPT	Non-surgical periodontal therapy
50.	NI	No information
51.	NA	Not applicable
52.	NS	Not specified
53.	PI	Plaque index
54.	P.i.	Prevotella intermedia
55.	P.g.	Porphyromonas gingivalis
56.	PBM	Photobiomodulation
57.	PGE 2	Prostaglandin E2
58.	PG	Parallel group
59.	PRISMA	Preferred Reporting Items for Systematic Reviews and Meta-Analyses
60.	PROSPERO	Prospective Register Of Systematic Reviews
61.	PPD/PD	Probing pocket depth
62.	PMN	Polymorphonuclear neutrophil
63.	qPCR	Quantitative polymerase chain reaction
64.	Q	Cochran’s heterogeneity statistic
65.	RAL	Relative attachment level
66.	RANKL	Receptor activator of nuclear factor kappa-B ligand
67.	RoB 2	Revised Cochrane Risk-of-Bias tool for Randomized trials, Version 2.0
68.	RCT	Randomized clinical trial
69.	RT-PCR	Reverse transcription polymerase chain reaction
70.	SRP	Scaling and Root planing
71.	SBI	Sulcus bleeding index
72.	SD	Standard deviation

73.	SM	Split mouth
74.	SE	Standard error
75.	SMD	Standardized mean difference
76.	SFFR	Sulcus fluid flow rate
77.	sec/ s	Second
78.	TBO	Toluidene blue O
79.	TNF-α	Tumor necrosis factor- alpha
80.	TDS	Three times daily
81.	T.f.	Tannerella forsythia
82.	T.d.	Treponema denticola
83.	UPD	Ultrasonic periodontal debridement
84.	VPI	Visual plaque index
85.	W	Watt
86.	W/cm^2	Watts per square centimeter
87.	Y	Yes
88.	%	Percentage
